# Reaction Suppression Between a High‐Ni Cathode Material (NMC622) and Li_7_La_3_Zr_2_O_12_ on Co‐Sintering for Manufacturing Bulk‐Type All‐Solid‐State Batteries: A New Method and Its Mechanism

**DOI:** 10.1002/advs.202512219

**Published:** 2025-08-29

**Authors:** Naohiro Hayashi, Ken Watanabe

**Affiliations:** ^1^ Hydrogen Business Development Div. DENSO CORPORATION Kariya Aichi 448‐8661 Japan; ^2^ Department of Advanced Materials Science and Engineering Faculty of Engineering Sciences Kyushu University Kasuga Fukuoka 816‐8580 Japan

**Keywords:** all‐solid‐state batteries, electrode/electrolyte interface, garnet, high‐nickel cathode materials

## Abstract

Co‐sintering cathode materials with Li_7_La_3_Zr_2_O_12_ (LLZ) is a promising strategy for fabricating bulk‐type all‐solid‐state batteries (ASSBs). However, preventing reactions between different materials, which is difficult with high‐capacity cathode materials such as LiNi_0.6_Mn_0.2_Co_0.2_O_2_ (NMC622), is a pre‐requisite for applying this strategy. To overcome this issue, Li_1+_ *_x_* Ni_0.6_Mn_0.2_Co_0.2_O_2_ (*x* = 0.01–0.2), which intentionally deviates from the stoichiometric NMC622 composition, is synthesized here. The formation of impurity phases in the co‐sintering process can be controlled by adjusting the co‐sintering temperature and *x*. Impurity phases are not formed on co‐sintering with *x* = 0.075 at 800 °C because reduced cation mixing in NMC622 and the presence of a self‐formed Li_2_CO_3_ layer on the particle surface, ensured by adjusting *x*, effectively suppresses reactions. Furthermore, good results are observed at sintering temperatures where the proportions of Ni^2+^ and Co^2+^, which promote cation mixing, are low. This study clarifies relevant reaction mechanisms using various analytical methods (such as temperature‐rise X‐ray absorption fine structure analysis and scanning transmission electron microscopy‐electron energy loss spectroscopy), and confirms the repetitive operation of bulk‐type ASSBs assembled using co‐sintered Li_1+_ *_x_* Ni_0.6_Mn_0.2_Co_0.2_O_2_ (*x* = 0.075)/LLZ electrolyte systems. The method reported herein can be potentially adopted for cost‐effective and high‐energy‐capacity ASSB production.

## Graphical abstract

A bulk‐type all‐solid‐state battery is produced by co‐sintering the proprietary NMC622 and LLZ. This proprietary NMC622 can avoid the formation of impurity phases under co‐sintering. The reduced Ni^2+^ fraction in NMC622 and the presence of a self‐formed Li_2_CO_3_ surface layer result in this outcome. It is possible to realize a rechargeable all‐solid‐state battery.

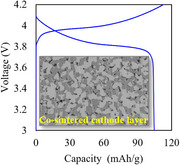

## 1 Introduction

Currently, the use of renewable energy is being promoted as a key step toward establishing a carbon‐neutral society. However, owing to high susceptibility toward climate change, the electricity supply from renewable energy sources is typically unstable. Consequently, high‐capacity Li‐ion batteries (LIBs) that can adjust to the fluctuations in renewable energy are in significant demand. Conventional LIBs are vulnerable to explosion hazards associated with the low flash points and flammability of organic electrolytes.^[^ ^1^, ^2^, ^3^ ^]^ To address this issue, nonflammable inorganic solid electrolytes are being used in all‐solid‐state LIBs (ASSBs), which are rapidly gaining attention for their high capacity and safety.^[^ ^4^ ^]^

Improving electrode properties, such as increasing the cathode voltage and anode capacity, is critical for the development of high‐capacity ASSBs. Therefore, the solid electrolytes used in ASSBs are required to exhibit high Li‐ion conductivity and a wide electrochemical window. Many types of solid electrolytes, including sulfides, halides, and oxides, have been studied over the past decades.^[^ ^5^, ^6^, ^7^, ^8^, ^9^, ^10^, ^11^ ^]^ Oxides exhibit acceptable chemical stability and good electrochemical oxidation stability. Among the oxide‐based solid electrolytes, garnet‐type Li_7_La_3_Zr_2_O_12_ (LLZ) is particularly promising because of its high stability against Li metal and relatively high ionic conductivity (>10^−4^ S cm^−1^) at 25 °C.^[^ ^12^, ^13^ ^]^

Although the ionic conductivity and electrochemical window of LLZ are sufficient, certain issues related to the formation of an electrolyte/electrode interface require resolution to enable the fabrication of high‐performance LLZ‐based ASSBs. The challenge of forming a good interface with the negative electrode, Li metal, can be overcome by inserting a coating layer that easily alloys with Li and soft electrolytes.^[^ ^14^, ^15^, ^16^, ^17^, ^18^ ^]^ However, forming a cathode material/LLZ interface suitable for battery operation remains a significant challenge. High‐temperature (>1000 °C) sintering is required to densify LLZ with high ionic conductivity. However, this process leads to the formation of a highly resistive interphase between the cathode material and LLZ, resulting in poor battery performance.^[^ ^19^, ^20^ ^]^ To solve this problem, numerous approaches for reducing the sintering temperature of LLZ, such as hot pressing, spark plasma sintering, and field‐assisted sintering technology, have been proposed to date.^[^ ^21^, ^22^, ^23^, ^24^, ^25^, ^26^, ^27^, ^28^ ^]^ However, these processes are expensive due to high equipment costs and batch processing. Therefore, current research is focused on investigating strategies to modify LLZ into a material that can be sintered at low temperatures.^[^ ^29^, ^30^, ^31^ ^]^ Ca–Bi–doped LLZ (LLZ‐CaBi) sintered at 750 °C with Li–Ca–Bi–O sintering aids shows a relative density of 94% and conductivity of 1.2 × 10^−3^ S cm^−1^.^[^ ^30^ ^]^ Therefore, an ASSB synthesized by co‐sintering LiCoO_2_ and LLZ‐CaBi can be operated for 80 cycles. LiCoO_2_ shows excellent thermal stability, making it a promising cathode material for ASSBs fabricated by co‐sintering.^[^ ^32^ ^]^ However, the use of LiCoO_2_ is limited by high material costs (owing to the presence of the rare element Co) and low capacity. Partially substituting the Co in LiCoO_2_ with other transition metals, such as Ni and Mn, results in the formation of LiNi*_x_* Mn*_y_* Co_1–_ *_x_*_–_ *_y_* O_2_ (NMC) cathode materials. High‐Ni NMC is superior to LiCoO_2_ in terms of cost and capacity, both of which are essential in high‐capacity ASSBs. However, with increasing Ni content, NMC easily reacts with LLZ at low sintering temperatures, preventing co‐sintering.^[^ ^33^, ^34^, ^35^, ^36^ ^]^ Multiple studies report the reaction onset temperature as well as La_2_Zr_2_O_7_ formation at a sintering temperature of 550 °C,^[^ ^36^ ^]^ indicating that these reactions can be prevented by lowering the sintering temperature of LLZ and improving its thermal stability.

The formation of a coating layer on the cathode material is widely used to suppress the decomposition reaction at the sulfide electrolyte/cathode material interface.^[^ ^37^, ^38^, ^39^, ^40^, ^41^, ^42^ ^]^ In LLZ electrolytes, coating layers comprising Li_3.5_Ge_0.5_V_0.5_O_4_, Nb_2_O_3_, and Li_2.985_B_0.005_OCl are known to suppress reactions with LiCoO_2_ during sintering.^[^ ^43^, ^44^, ^45^ ^]^ Conventional co‐sintering with NMC remains unreported to date, remaining a major technical challenge in ASSB research.

This study focuses on the co‐sintering of Li_0.6_Mn_0.2_Co_0.2_O_2_ (NMC622) and LLZ‐CaBi. NMC622 was considered for use in ASSBs owing to its widespread utilization in conventional LIBs. Li‐excess NMC622 (Li_1+_ *_x_* Ni_0.6_Mn_0.2_Co_0.2_O_2_) was used for co‐sintering. In Li_1+_ *_x_* CoO_2_, excess Li is reported to spontaneously form a Li_2_CO_3_ coating layer,^[^ ^45^ ^]^ resulting in a cost‐effective coating process that does not require additional processing. The Li_2_CO_3_ coating layer, which is expected to prevent direct electrode/electrolyte contact. Also, *x* in Li_1+_ *_x_* CoO_2_ affects the Li‐occupied sites as well as the amount and orientation of oxygen defects in the system.^[^ ^46^, ^47^, ^48^ ^]^ Similar changes are expected to occur in Li‐excess‐type NMC622, leading to changes in its reactivity toward LLZ‐CaBi. This study aims to clarify the effect of excess Li on the reactivity of NMC622 with LLZ‐CaBi along with the underlying mechanism of this process. After analysis, a co‐sintered bulk‐type ASSB was manufactured using the developed NMC622 powder.

## 2 Results and Discussion

### 2.1 Characterization and Reactivity of L_1+_ *_x_* Ni_0.6_Mn_0.2_Co_0.2_O_2_ Powders

Table S1 (Supporting Information) shows the results of compositional analysis using inductively coupled plasma atomic emission spectroscopy (ICP–AES). The molar ratio was calculated based on Co. The tabulated data indicate that the amount of Li increases with increasing *x*. However, the actual Li ratio in the particles tends to be lower than the Li ratio targeted during synthesis, likely due to Li volatilization during high‐temperature synthesis.^[^ ^49^ ^]^

**Figure 1:**
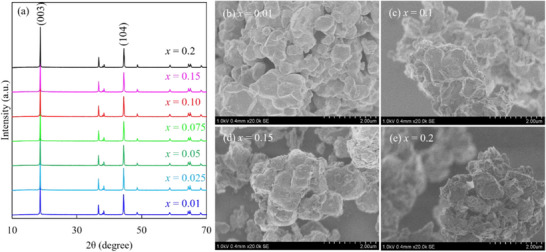
a) Powder X‐ray diffraction patterns of the Li_1+_ *_x_* Ni_0.6_Mn_0.2_Co_0.2_O_2_ powder synthesized in this study. Scanning electron microscopy images of Li_1+_ *_x_* Ni_0.6_Mn_0.2_Co_0.2_O_2_ particles at *x* = b) 0.01, c) 0.1, d) 0.15, and e) 0.2.

**Figures** **1** and S1 (Supporting Information) show the powder X‐ray diffraction (XRD) patterns and scanning electron microscopy (SEM) images of Li_1+_ *_x_* Ni_0.6_Mn_0.2_Co_0.2_O_2_ (*x* = 0.01–0.2). Figure 1a indicates that the spectral peaks of all samples are identical to those of pure NMC622. Moreover, the XRD patterns correspond to the α‐NaFeO_2_ structure with the space group *R3m*.^[^ ^50^ ^]^ Figure S1 (Supporting Information) shows that both the (006)/(012) and (018)/(110) doublets are split, indicating a well‐crystallized layered‐structure material. The analyzed lattice constant refinement results (summarized in Table S2, Supporting Information) indicate that the lattice parameters decrease with increasing *x*. This phenomenon, which is attributable to an increase in the oxidation numbers of Ni, Mn, and Co, is described in detail later. In Figure 1b, clear facets are observed on the particle surface of the *x* = 0.01 sample. However, with increasing *x*, deposits are clearly observed on the particle surface (Figure 1c–e). Notably, the particle size (≈1.5 µm) is independent of *x*.

**Figure 2:**
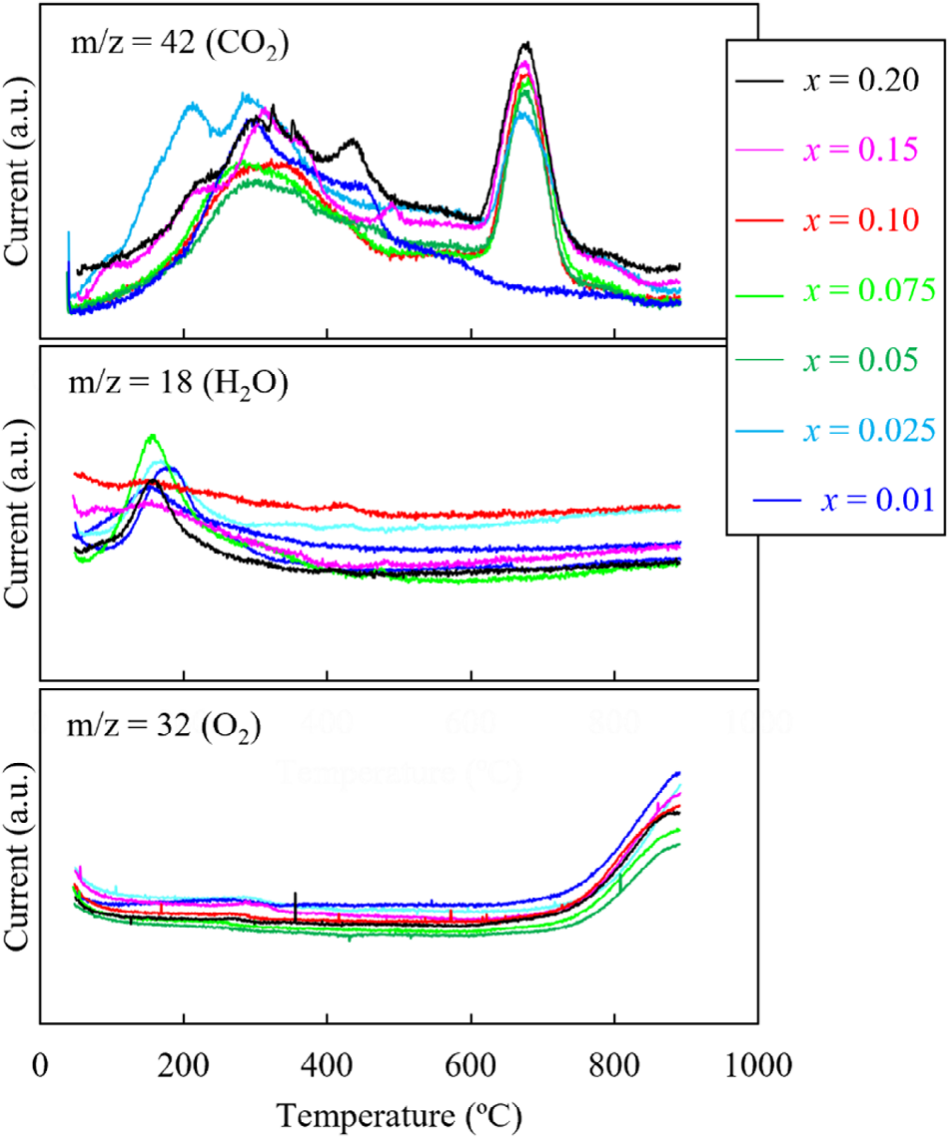
Gas chromatography–mass spectrometry spectra of the Li_1+_ *_x_* Ni_0.6_Mn_0.2_Co_0.2_O_2_ particles.

**Figure** **2** shows the temperature dependence of the gas chromatography–mass spectrometry (GC–MS) patterns of the synthesized samples. All the samples show CO_2_ and H_2_O desorption at temperatures below 530 °C. According to Sickiliger et al., Ni‐rich cathode materials easily undergo NiCO_3_·2Ni(OH)_2_·*x*H_2_O contamination, resulting in weight loss.^[^ ^51^ ^]^ In this study, samples with *x* ≥ 0.05 release CO_2_ at ≈ 680 °C. The melting point of Li_2_CO_3_ is 723 °C, which is lowered in the presence of NiO.^[^ ^52^ ^]^ Excess Li produces a Li_2_CO_3_‐like phase that decomposes within this time. With increasing *x*, the amount of CO_2_ released increases slightly, indicating an increase in Li_2_CO_3_ production. Therefore, the deposits shown in Figure 1c–e are likely Li_2_CO_3_. Table S3 (Supporting Information) summarizes the amount of Li_2_CO_3_ calculated based on the TG–DTA data of the sample with *x* = 0.2 in Figure S2 (Supporting Information). When *x* = 0.025–0.15, these values are calculated from the ratio to *x* = 0.2 in the GC–MS results. Oxygen desorption is observed from ≈720 °C. Under Ar, LiNi_0.8_Mn_0.1_Co_0.1_O_2_ (NMC811) and LiNi_1/3_Mn_1/3_Co_1/3_O_2_ (NMC111) decompose at 675 and 825 °C, respectively, accompanied with oxygen release.^[^ ^51^ ^]^ This decomposition behavior is consistent with the thermal stability of partially delithiated Ni‐rich cathode materials. According to Noh et al., the thermal stability of NMC622 is intermediate between those of NMC111 and NMC811; therefore, the NMC622 decomposition temperature observed in this study is reasonable.^[^ ^53^ ^]^

**Figure 3:**
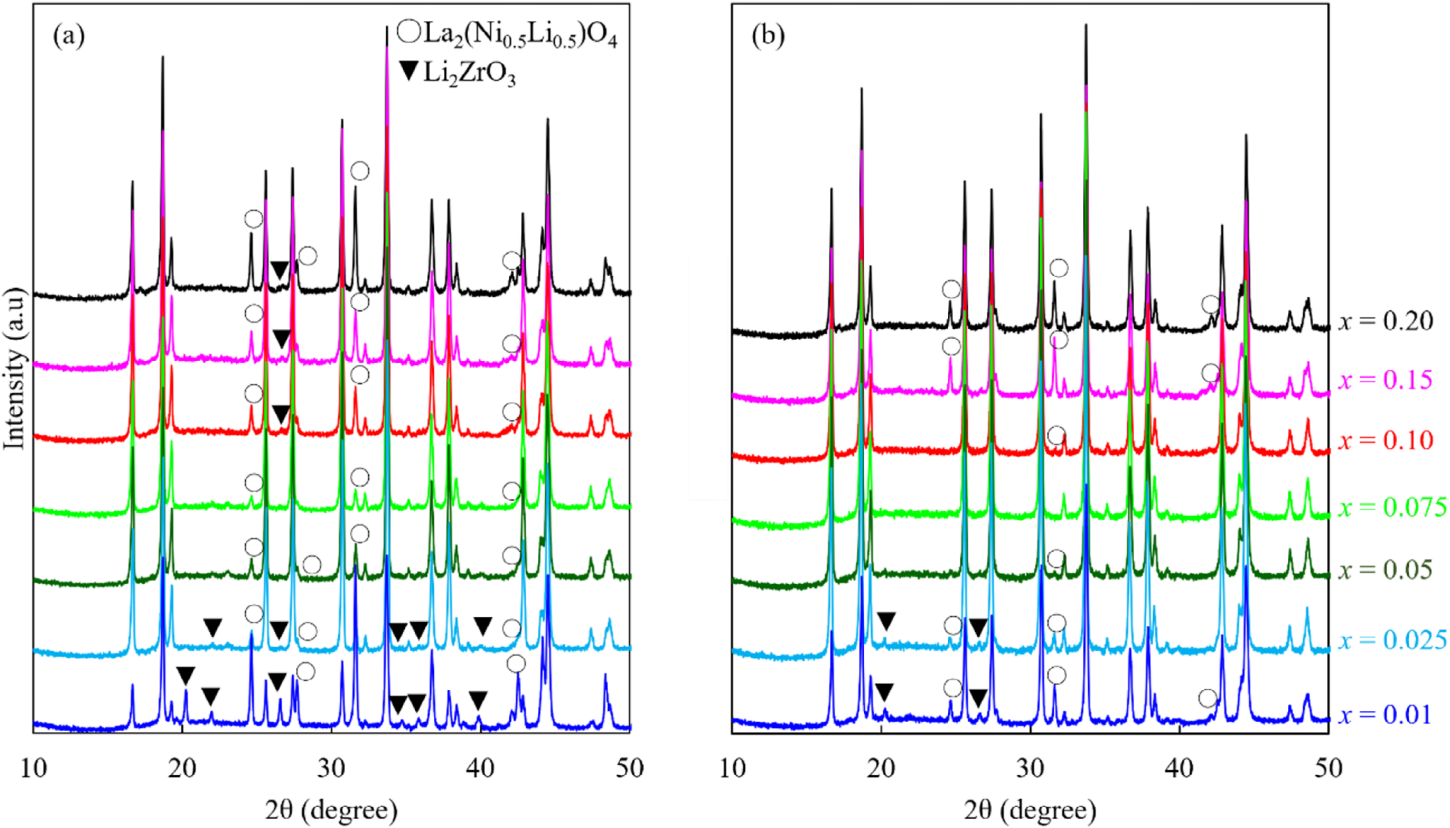
Powder X‐ray diffraction spectra of the Li_1+_ *_x_* Ni_0.6_Mn_0.2_Co_0.2_O_2_ (*x* = 0.01–0.2) and LLZ‐CaBi mixture powders after sintering at a) 750 and b) 800 °C.

**Figure** **3** shows the reactivity results of co‐sintering with LLZ‐CaBi and Li_1+_ *_x_* Ni_0.6_Mn_0.2_Co_0.2_O_2_ (*x* = 0.01–0.2). When co‐sintered at 750 °C, impurity phases are observed at all *x* values (Figure 3a). The spectral results indicate that La(Ni_0.5_Li_0.5_)O_4_ is formed at all *x* values, whereas Li_2_ZrO_3_ is formed only at *x* = 0.01, 0.025, 0.10, 0.15, and 0.20. According to Roitzheim et al., co‐sintering NMC811 and Li_6.45_Al_0.05_La_3_Zr_1.6_Ta_0.4_O_12_ within 650–1000 and 550–650 °C results in the formation of La(Ni_0.5_Li_0.5_)O_4_ and La_2_Zr_2_O_7_, respectively.^[^ ^36^ ^]^ In addition, according to Ohta et al., La(Ni_0.5_Li_0.5_)O_4_ is formed when NMC111 and Li_6.4_Al_0.2_La_2.25_Ca_0.75_Zr_1.25_Nb_0.75_O_12_ are co‐sintered at 750 °C.^[^ ^54^ ^]^ Therefore, the results of this study are consistent with the literature. The amount of impurities decreases with increasing *x* when *x* ≤ 0.075, whereas it increases with increasing *x* when *x* > 0.075. Significant Li_2_ZrO_4_ formation is observed when the amount of the main impurity phase, i.e., La(Ni_0.5_Li_0.5_)O_4_, is high. The reactivity at 800 °C is shown in Figure 3b. Surprisingly, a lower amount of impurities is observed at 800 °C than at 750 °C, likely because elemental diffusion increases at higher temperatures. According to Scheld et al. and Ohta et al., the reactivity of LLZ with the cathode material varies with the doping element.^[^ ^54^, ^55^ ^]^ The results of this study indicate that the sintering temperature may affect the reactivity of the cathode with LLZ and suggest an optimal temperature to avoid the formation of impurities. The relevant mechanism is discussed later. Interestingly, the dependence of *x* on the amount of impurities shows the same tendency for co‐sintering at 750 and 800 °C. Consequently, no impurity phase is formed for *x* = 0.075 at 800 °C. Thus, the reactivity of NMC622 and LLZ‐CaBi can be controlled by tuning *x* and the sintering temperature.

### 2.2 Investigating the Mechanism of Reaction Inhibition by Li_1+_ *_x_* Ni_0.6_Mn_0.2_Co_0.2_O_2_

#### 2.2.1 Local‐Structure Analysis of Li_1+x_Ni_0.6_Mn_0.2_Co_0.2_O_2_ by X‐Ray Absorption Fine Structure (XAFS) Analysis

Figure S3a–c (Supporting Information) show the normalized Ni, Mn, and Co K‐edge XANES spectra of Li_1+_ *_x_* Ni_0.6_Mn_0.2_Co_0.2_O_2_ (*x* = 0.01–0.2). In the Ni K‐edge spectra (Figure S3a, Supporting Information), the edges shift to higher energies with increasing *x*, indicating that Ni transitions to a more oxidized state. In contrast, the Mn and Co edges do not change significantly with increasing *x* (Figure S3b,c, Supporting Information).

**Figure 4:**
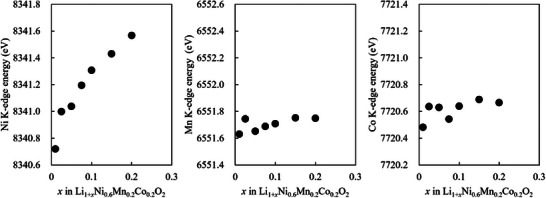
Dependence of the Ni, Mn, and Co K‐edge spectra of Li_1+_ *_x_* Ni_0.6_Mn_0.2_Co_0.2_O_2_ on *x*.

**Figure** **4** shows the dependence of the Ni‐, Mn‐, and Co‐edge energies on *x*. With increasing *x*, the Ni K‐edge shifts significantly toward higher energies, whereas the Mn‐ and Co‐edge show relatively slight shifts toward higher energies. According to the literature, NMC622 contains an Ni^2+^/Ni^3+^ ratio of 1:2 (i.e., an average of Ni^2.67+^).^[^ ^56^, ^57^, ^58^, ^59^ ^]^ Therefore, the proportion of Ni^3+^ is expected to increase with increasing *x*. Figure S4 (Supporting Information) summarizes the edge energies of the Mn and Co reference samples measured at the same beamline. Here, Mn^4+^ and Co^3+^ were observed in NMC622, consistent with a previous study.

**Figure 5:**
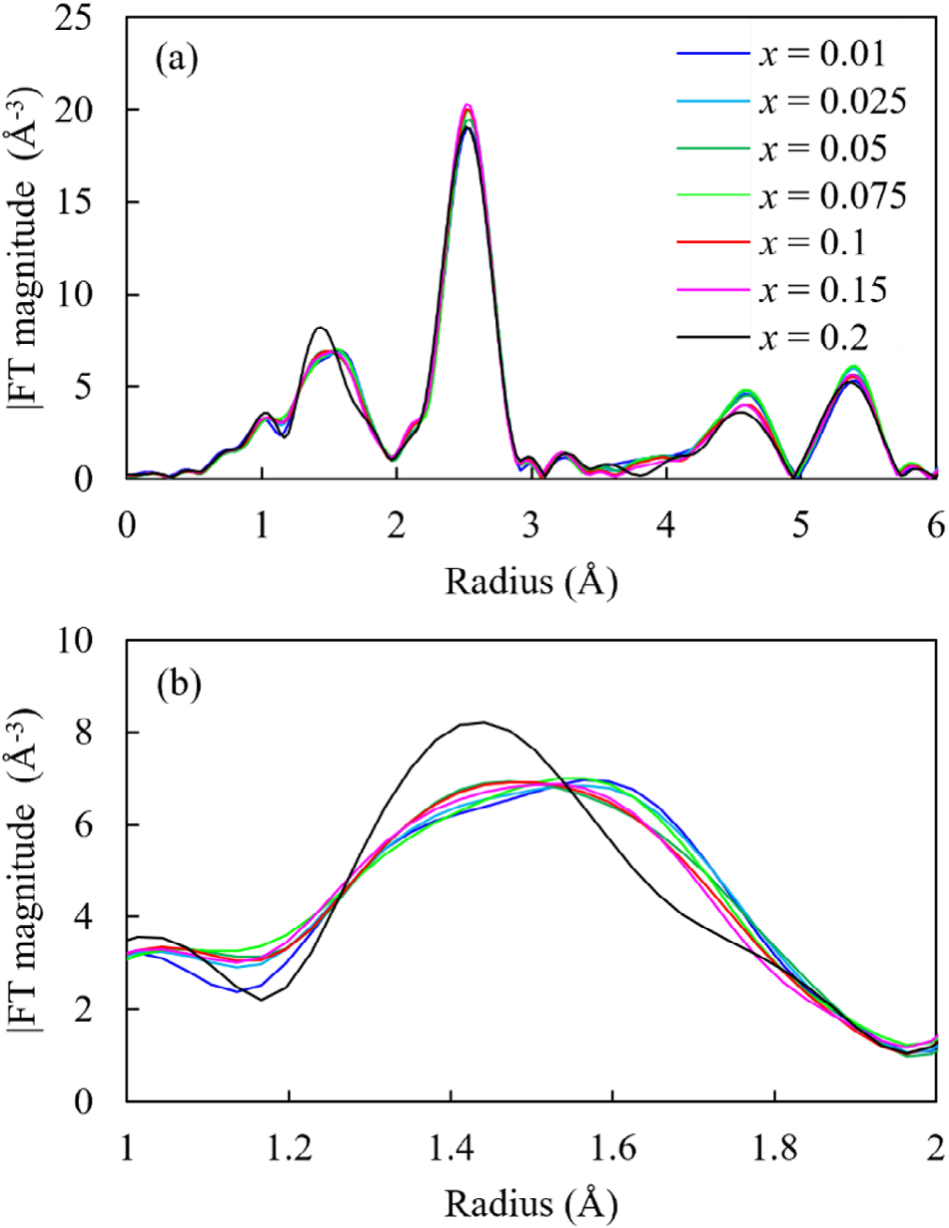
a) Extended X‐ray absorption fine structure spectra and b) enlarged view of the Ni–O peak in the Ni K‐edge spectra of the synthesized samples.

**Figure 6:**
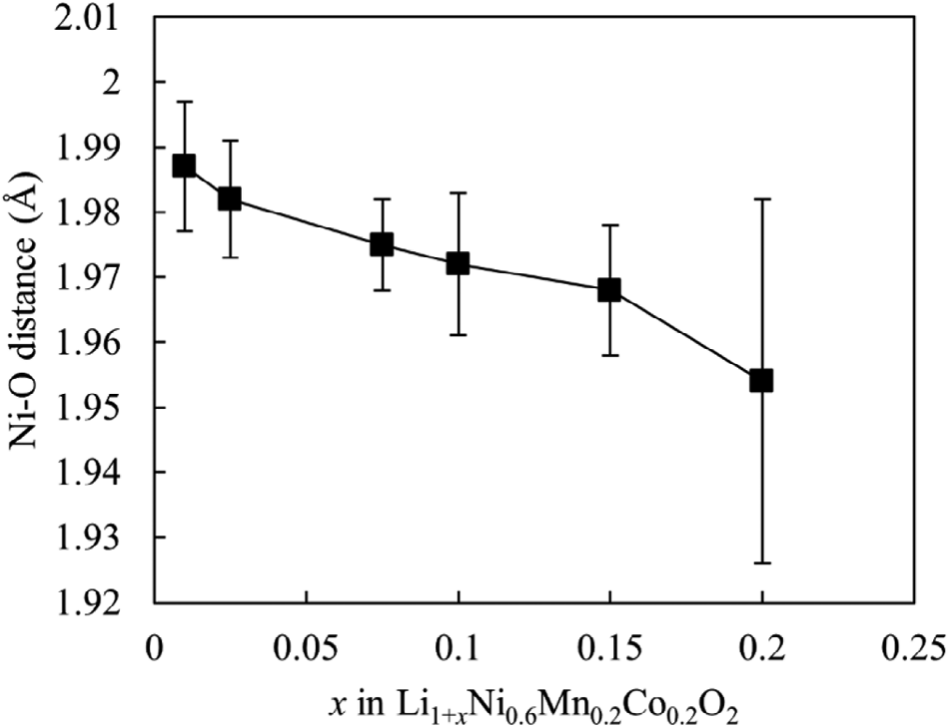
Dependence of the Ni─O bond length in Li_1+x_Ni_0.6_Mn_0.2_Co_0.2_O_2_ on *x*.

The radial structure function of the Ni K‐edge is shown in **Figure** **5**, and those of the Mn and Co K‐edge are shown in Figure S5 (Supporting Information). Compared with the extended X‐ray absorption fine structure (EXAFS) spectra of the Mn and Co K‐edge, the EXAFS spectra of the Ni K‐edge change significantly with *x*. In the Ni K‐edge spectra, the first and second peaks (≈1.5 and ≈2.5 Å, respectively) can be attributed to Ni–O and Ni–M interactions, respectively (M represents transition metals such as Ni, Mn, and Co). With an increase in *x* (up to 0.075), the Ni–O peak tends to broaden, likely owing to the Jahn–Teller distortion of d^7^ Ni^3+^. The Jahn–Teller distortion, characterized by Ni–ligand bond lengths becoming inequivalent, results in the widening of the Ni–O peak. Consequently, two types of Ni–O distances (long and short) exist within the system, leading to a distortion of the Ni–O octahedron. At *x* = 0.2, the Ni–O peak becomes sharper with an increase in intensity. This suggests that the oxidation state of Ni is greater than the trivalent state, resulting in Jahn–Teller distortion. **Figure** **6** shows the Ni─O bond length obtained by fitting the EXAFS spectra. The bond length decreases with increasing *x*. This phenomenon can be attributed to a change in the effective nuclear charge, which increases with increasing oxidation state. This change in the oxidation state of Ni affects the local structure around Mn and Co. In the spectra shown in Figure S5 (Supporting Information), the second peak corresponds to Mn–M and Co–M; because Ni occupies ≈60% of M, the second peak changes with changes in *x*.

#### 2.2.2 Surface‐Structure Analysis of Li_1+x_Ni_0.6_Mn_0.2_Co_0.2_O_2_ Using Scanning Transmission Electron Microscopy–Electron Energy Loss Spectroscopy (STEM–EELS)

**Figure 7:**
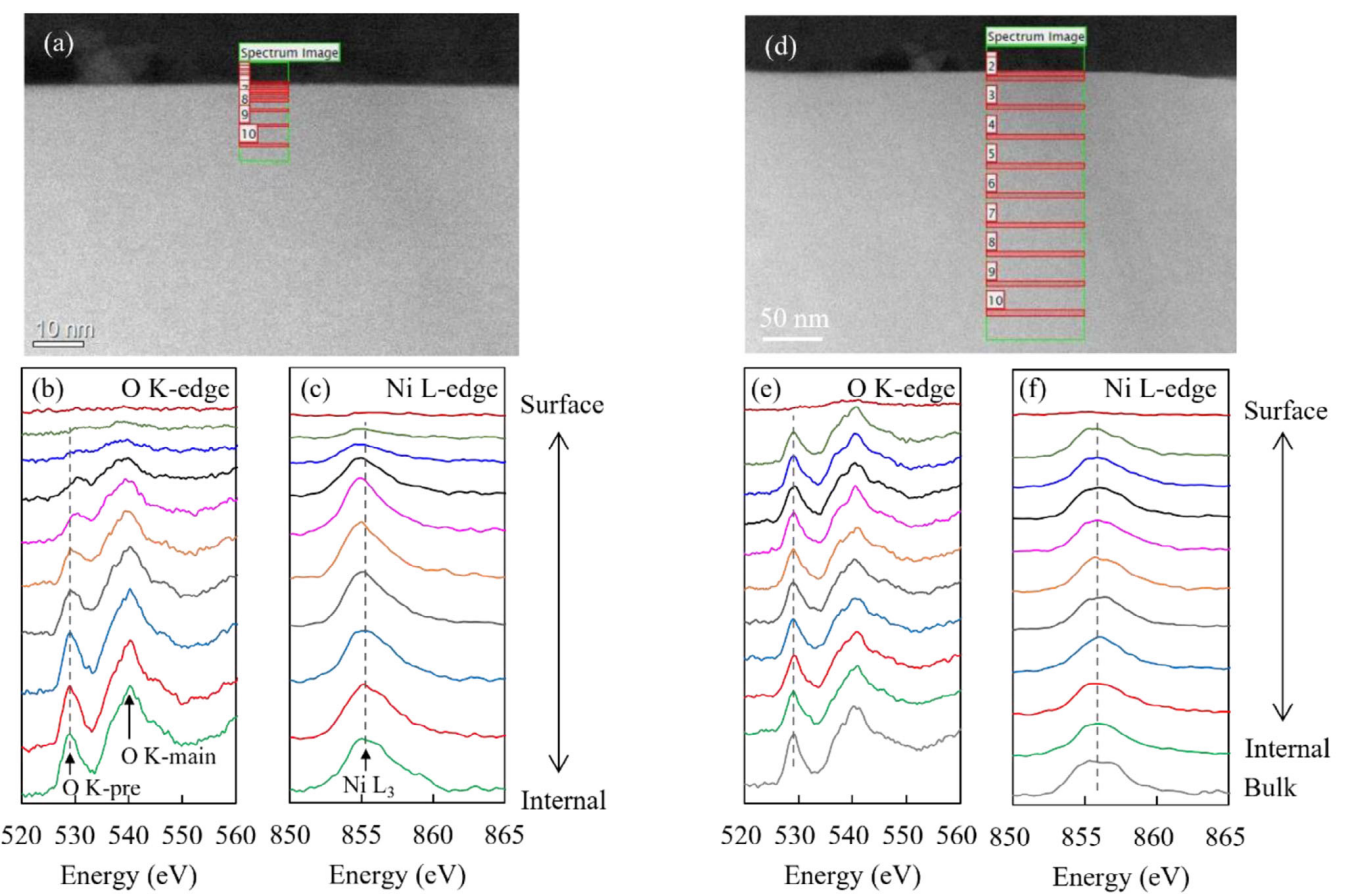
a) STEM–HAADF image as well as b) O K‐edge and c) Ni L‐edge electron energy loss spectroscopy patterns of the Li_1.01_Ni_0.6_Mn_0.2_Co_0.2_O_2_ particles (from the surface to the interior). b) STEM–HAADF image as well as c) O K‐edge and d) Ni L‐edge electron energy‐loss spectroscopy patterns of the Li_1.075_Ni_0.6_Mn_0.2_Co_0.2_O_2_ particles (from the surface to the interior).

**Figure** **7** and Figures S6 and S7 (Supporting Information) show the STEM image and EELS spectra for the Li K‐edge, O K‐edge, Ni L‐edge, Mn L‐edge, and Co L‐edge of the Li_1+_ *_x_* Ni_0.6_Mn_0.2_Co_0.2_O_2_ (*x* = 0.01) particle surfaces. On approaching the particle surface, the pre‐edge of the O K‐edge shifts toward higher energies, while the L‐edges of Ni, Mn, and Co shift toward lower energies. In Ni‐rich cathode materials, the crystal structure of the particle surface can differ from that of the interior in the initial state and after cycling.^[^ ^49^, ^60^, ^61^ ^]^ According to Park et al., changes are observed in the EELS spectrum of LiNiO_2_ upon cation mixing, and the system is transformed into the rock‐salt structure.^[^ ^60^ ^]^ On cation mixing, the pre‐edge of the O K‐edge shifts to higher energies. On transformation to the rock‐salt structure, a shift occurs toward higher energies, and the pre‐edge and main edge do not remain clearly split, indicating the formation of oxygen vacancies. According to the literature, the oxidation state of transition metals decreases with increasing generation of oxygen vacancies.^[^ ^62^ ^]^ Moreover, Li^+^ (0.76 Å) and Ni^2+^ (0.69 Å), with similar ionic radii, can undergo mixing.^[^ ^49^ ^]^ Based on the literature, oxygen vacancies were assumed to be generated on the particle surface and cation mixing was assumed to occur at *x* = 0.01.

**Figure 8:**
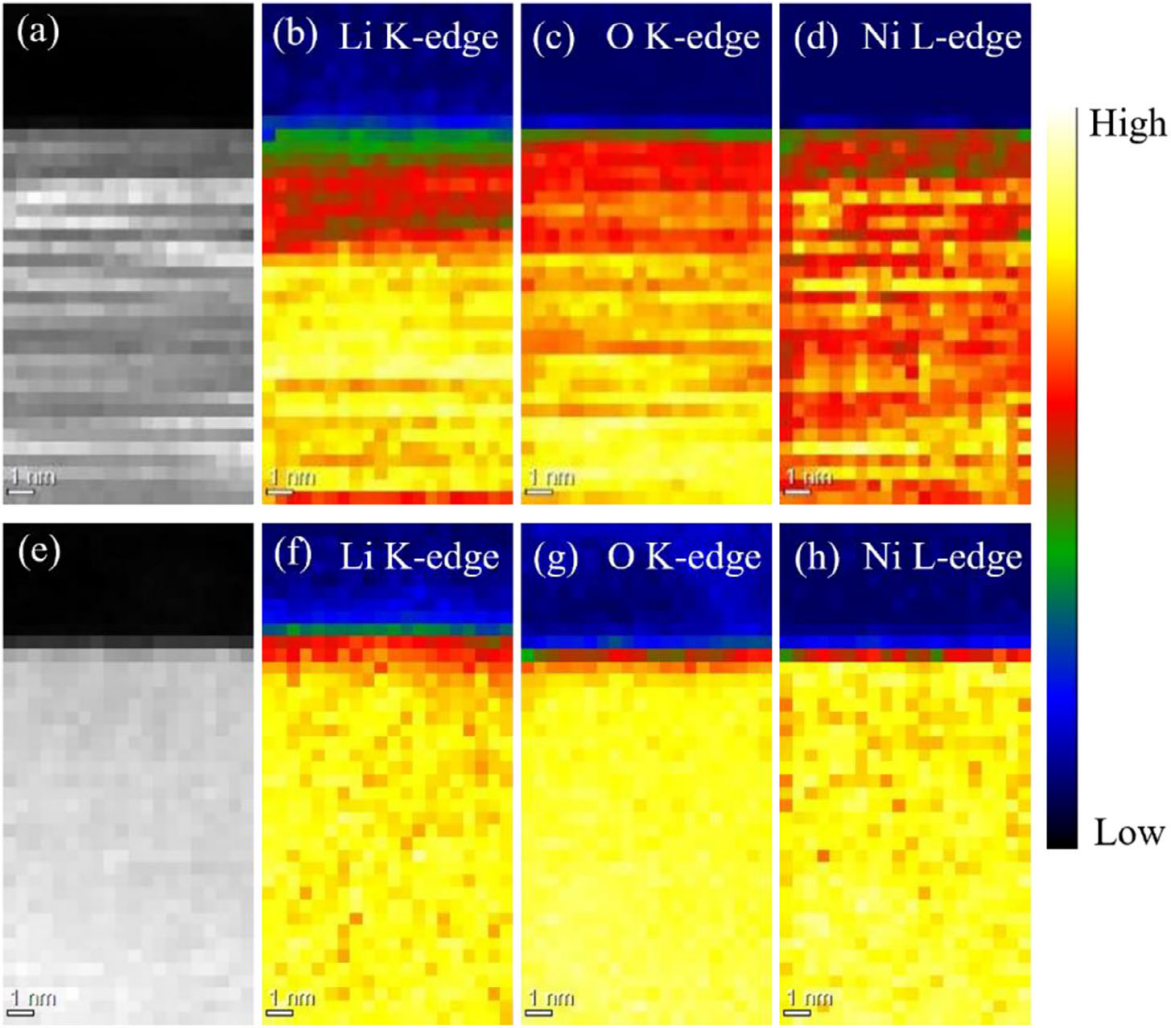
Electron energy loss spectroscopy mapping of the particles surface for a–d) Li_1.01_Ni_0.6_Mn_0.2_Co_0.2_O_2_ and e–h) Li_1.075_Ni_0.6_Mn_0.2_Co_0.2_O_2_. As the Li K‐edge overlaps with the Co and Ni M‐edge, the distribution is determined by focusing on the peak intensity ≈62 eV.

The EELS spectra of the Li_1+_ *_x_* Ni_0.6_Mn_0.2_Co_0.2_O_2_ (*x* = 0.075) particle surface and bulk (the center of the particle is ≈265 nm from the surface) are shown in **Figures** **8** and S6 (Supporting Information). Interestingly, the EELS spectra (O, Ni, Mn, and Co edges) of the interior and surface of the particles are similar. At *x* = 0.075, some oxygen deficiency is observed on the particle surface along with a low degree of cation mixing. Notably, for the interior and surface of the particles with *x* = 0.01, the peak top of the Ni L_3_‐edge is located at the high‐energy side, indicating a high proportion of Ni^3+^. This is consistent with the results shown in Figure 4.

Figure 8 shows the EELS mapping of the particle surface for Li_1.01_Ni_0.6_Mn_0.2_Co_0.2_O_2_ and Li_1.075_Ni_0.6_Mn_0.2_Co_0.2_O_2_. Compared with the particle with *x* = 0.01, the surface of the particle with *x* = 0.075 contains a thinner region with lower Li and O concentrations than the interior. This distribution suggests that the particles with *x* = 0.075 contain fewer Li and oxygen defects on the surface and less cation mixing than those with *x* = 0.01. The intensity ratio I(003)/I(104) from the XRD patterns of the samples was used to quantify the degree of cation mixing. A higher value of I(003)/I(104) implies a lower degree of cation mixing.^[^ ^53^ ^]^ The I(003)/I(104) values listed in Table S4 (Supporting Information), calculated from the XRD pattern shown in Figure 1, indicate that the extent of cation mixing reduces with increasing *x*.

The above‐mentioned results indicate that increasing *x* results in an effective suppression of Li^+^/Ni^2+^ cation mixing throughout the particle along with a reduction in the Li and oxygen deficiency and cation mixing between the transition metals (Ni, Mn, and Co) and Li^+^ on the particle surface. These changes and the presence of Li_2_CO_3_ likely cause a change in the reactivity of the electrode material with LLZ‐CaBi.

#### 2.2.3 Local‐Structure Analysis of Li_1+x_Ni_0.6_Mn_0.2_Co_0.2_O_2_ by High‐Temperature XAFS Analysis

**Figure 9:**
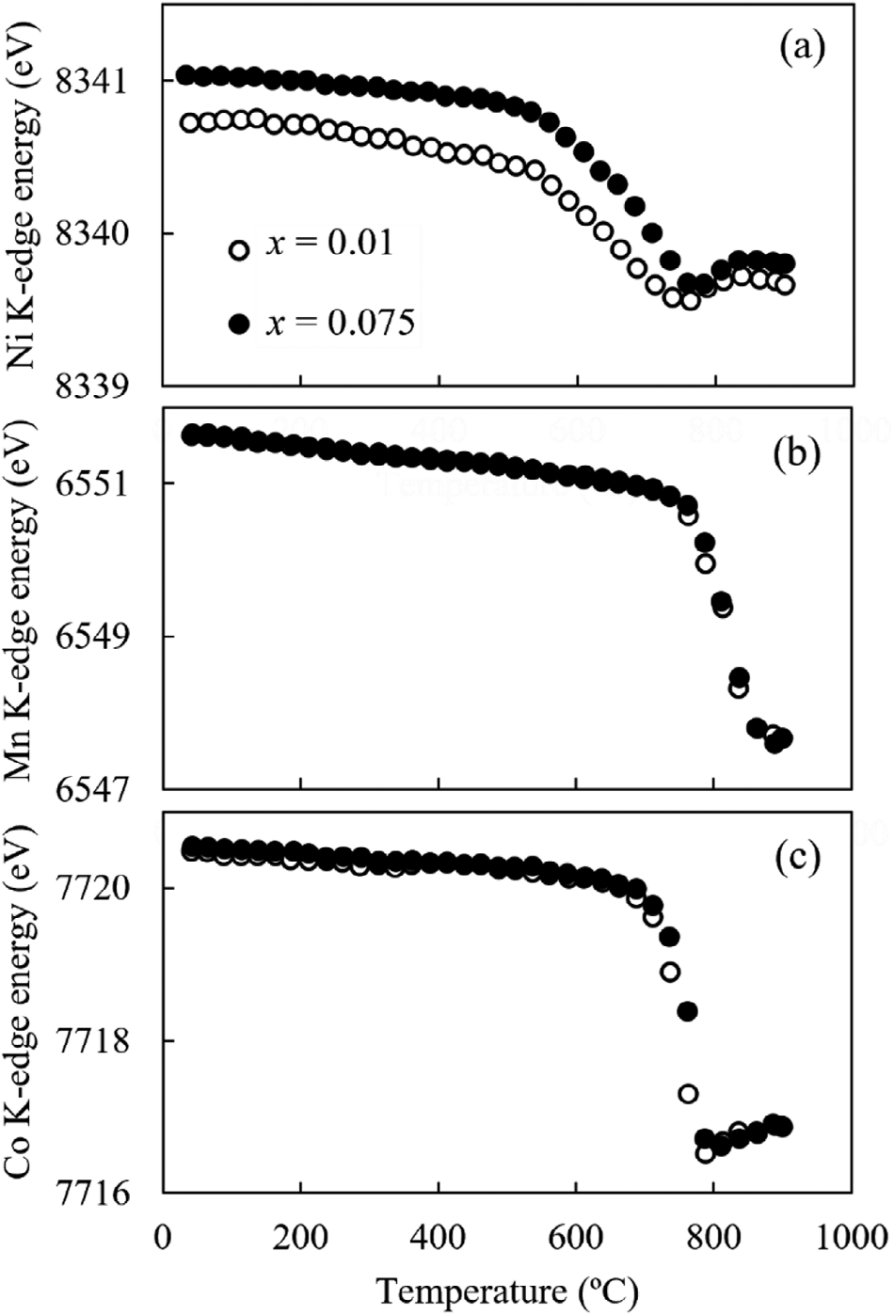
Temperature dependence of the a) Ni, b) Mn, and c) Co K‐edge energies for samples with *x* = 0.01 and 0.075.

As mentioned in the previous section, a greater suppression in impurity phase formation is observed on sintering at 800 °C than at 750 °C. High‐temperature XANES measurements were used to investigate this phenomenon. The temperature dependence of the Ni, Mn, and Co K‐edge energies is shown in **Figure** **9**. The Ni K‐edge energy begins to shift to lower energies at lower temperatures compared to Co, Mn K‐edge. Initially (from ≈ 540 °C), the Ni K‐edge energy shifts abruptly to the low‐energy side; subsequently, after showing the lowest value at ≈ 760 °C, the energy shifts again to the high‐energy side (Figure 9a). Figure 9b,c shows that a shift to lower energies occurs at ≈ 760 and 710 °C for the Mn and Co K‐edge, respectively; subsequently, the Co K‐edge shifts to higher energies at ≈ 780 °C. Based on the oxygen desorption behavior shown in Figure 2, the behavior of the sample during the sintering process can be predicted; this is described next. Within 540–710 °C, where oxygen desorption is not clearly observed, the oxidation state of Ni decreases significantly. At ≈ 710–760 °C, where gradual oxygen desorption begins, the oxidation states of Ni and Co decrease. Above 760 °C, where oxygen desorption occurs rapidly, the oxidation state of Mn decreases rapidly, whereas those of Ni and Co increase. Thus, the oxidation states of Ni, Mn, and Co change in a complex manner with oxygen desorption. Ni and Co exhibit low oxidation states at 750 °C, which increase at 800 °C. Therefore, cation mixing is highly probable at 750 °C, and holding the material at this temperature for a prolonged duration might lead to the formation of an impure phase.

These results indicate that the transition‐metal oxidation state and oxygen desorption significantly affect the reactivities of NMC622 and LLZ‐CaBi. However, the behavior of cathode materials at high temperatures that are not used in conventional LIBs remains largely unknown to date and requires further investigation.

#### 2.2.4 Analysis of the Li_1+x_Ni_0.6_Mn_0.2_Co_0.2_O_2_/LLZ‐CaBi Interface After Sintering

**Figure 10:**
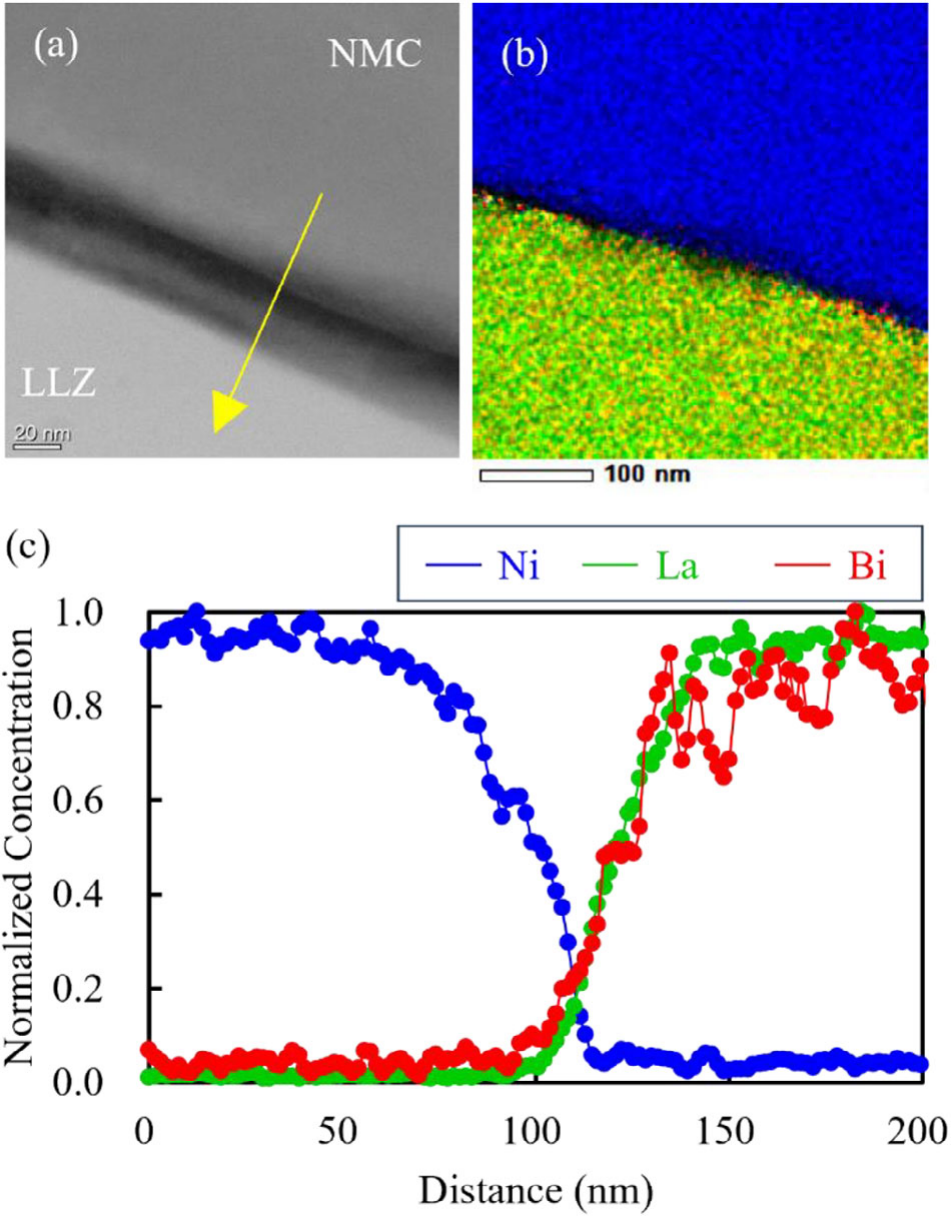
a) STEM–HAADF image, b) energy‐dispersive X‐ray spectroscopy superposition mapping (blue: Ni, green: La, red: Bi), and c) element concentration profiles of the NMC622 (x = 0.075)/LLZ‐CaBi interface produced by sintering at 800 °C.

**Figure 11:**
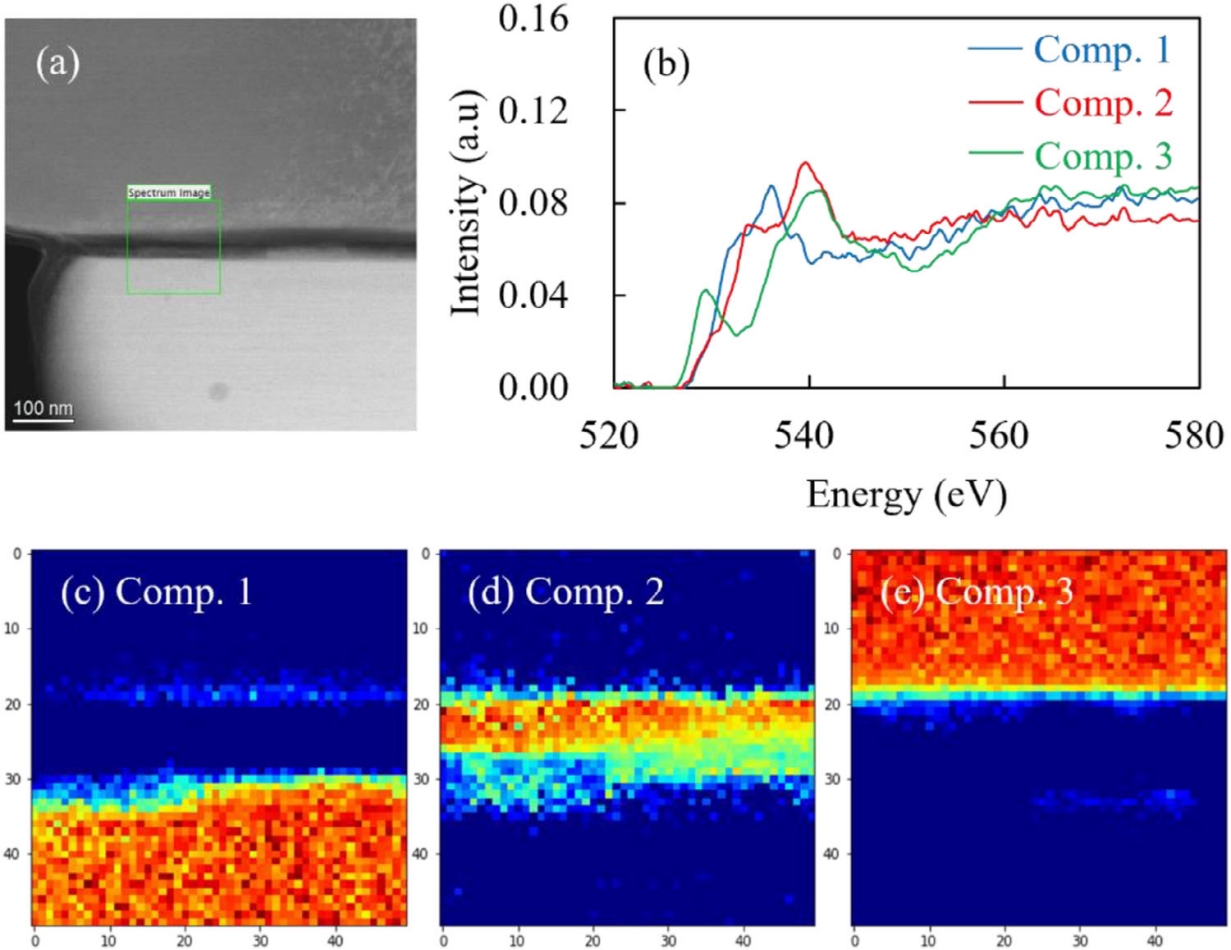
a) STEM–HAADF image, b) spectrum after decomposition by multivariate analysis, and c–e) imaging results for each component of the NMC622 (x = 0.075)/LLZ‐CaBi interface produced by sintering at 800 °C.

The STEM–HAADF image, energy‐dispersive X‐ray spectroscopy (EDX) mapping, and line profile at the interface between NMC622 (*x* = 0.075) and LLZ‐CaBi after sintering at 800 °C are shown in **Figure** **10**. An ≈20 nm‐thick layer with different brightness values is observed at the interface (Figure 10a), indicating that the interface layer is composed of two layers that exhibit two different brightness levels. The Ni, La, and Bi EDX maps are superposed in Figure 10b. The interfacial layer close to the NMC622 particle surface is dark; consequently, the elemental composition of this layer could not be confirmed. However, Bi is confirmed to be concentrated at the interface‐layer region close to the LLZ‐CaBi particle surface. This is validated by Figure 10c, which shows that the Bi profile is shifted by ≈10 nm toward the NMC particle side compared with the La profile. Figure S8 (Supporting Information) shows the results of a similar analysis for the NMC622 (*x* = 0.01)/LLZ‐CaBi interface. In this case, two interfacial layers with different brightness values cannot be identified (Figure 10), and a single layer is observed. Figure S8b,c (Supporting Information) indicate the presence of a single Bi‐enriched layer (≈4 nm thick) at the interface. EELS was used to analyze this interface layer in greater detail. EELS spectral data were used for multivariate analysis based on O K‐edge data, and the superimposed spectra were separated and visualized for each component (**Figure** **11**). Components 1 and 3 represent LLZ‐CaBi and NMC622, respectively. The spectral shape of component 2 differs from those of components 1 and 3. Similar data for the NMC622 (*x* = 0.01)/LLZ‐CaBi interface are shown in Figure S9 (Supporting Information). Although this interface is thinner than that formed with NMC (*x* = 0.075), both interfaces contain component 2. Figure S10 (Supporting Information) compares the O and Li K‐edge data of the samples with those of the reference Li_2_O; similar spectral shapes suggest that component 2 is a Li_2_O‐based layer. According to the literature, LLZ‐CaBi self‐generates a Li–Ca–Bi–O liquid phase.^[^ ^30^ ^]^ Therefore, a Bi‐enriched Li_2_O‐based interface layer is likely formed in NM622 (*x* = 0.01, 0.075). In NMC622 (*x* = 0.075), the self‐generated Li_2_CO_3_ layer on the surface decomposes to form a Li_2_O layer. Owing to the formation of two types of interface layers, the interface layer generated for NMC622 (*x* = 0.075) is thicker than that generated for NMC622 (*x* = 0.1). According to Nolan et al., Li_2_O is an effective coating layer that prevents reactions between LLZ and the cathode material.^[^ ^63^ ^]^ The results of this study indicate that a Li_2_O‐based interface layer of appropriate thickness is formed at the NMC622 (*x* = 0.075)/LLZ‐CaBi interface, resulting in the inhibition of electrode/electrolyte reactions.

### 2.3 NMC622/LLZ‐CaBi Reaction and Inhibitory Mechanism

Based on the results of analyses described in previous sections, the reaction scheme for the co‐sintering of NMC622 (*x* = 0.01) and LLZ‐CaBi is proposed here. The reaction scheme can be divided into the following sections: the initial state, 540–710 °C sintering stage, 710–760 °C sintering stage, and 760–800 °C sintering stage. The phenomena occurring in each section are described next.

Initial state: Ni exists in a mixed state (comprising both Ni^2+^ and Ni^3+^) with an average oxidation state of 2.67, Co exists in the Co^3+^ state, and Mn exists in the Mn^4+^ state throughout the NMC622 particles. Abundant oxygen vacancies, a Li deficiency, and low‐valence transition metals (such as Ni^2+^, Co^2+^, Mn^3+^) are observed on the particle surface, resulting in cation mixing between Li^+^ (0.76 Å) and Ni^2+^ (0.69 Å), Co^2+^ (0.65Å).

540–710 °C sintering stage: A change from Ni^3+^ to Ni^2+^ occurs along with significant cation mixing with Li^+^. Moreover, owing to similar ionic radii, the Li^+^ and Ni^2+^ in NMC622 and LLZ‐CaBi undergo vigorous mixing at high temperatures. Owing to the difference in valence, a large amount of Li^+^ is displaced from LLZ‐CaBi, resulting in significant Li depletion within the LLZ‐CaBi structure. Consequently, LLZ‐CaBi is rendered unstable and decomposes into La(Ni_0.5_Li_0.5_)O_4_ and Li_2_ZrO_3_.

710–760 °C sintering stage: Certain transformations (Ni^3+^ to Ni^2+^ and Co^3+^ to Co^2+^) occur on gradually increasing the oxygen vacancies in NMC622. Among all the tested temperatures, the highest populations of Ni^2+^ and Co^2+^ are observed within this temperature range, resulting in extremely frequent cation mixing. Therefore, maintaining the sintering temperature within this range for a long time promotes the formation of La(Ni_0.5_Li_0.5_)O_4_ and Li_2_ZrO_3_.

760–800 °C sintering stage: NMC622 loses a large amount of oxygen; simultaneously, Mn^4+^ is reduced to Mn^3+^, while the valence of Ni^2+^ and Co^2+^ is increased. Since the ionic radius of Mn^3+^ (0.58 Å) is not as close to Li^+^ as those of Ni^2+^ and Co^2+^, cation mixing is unlikely. As the migration of Ni^2+^ and Co^2+^ to LLZ‐CaBi is reduced, the formation of impurity phases is challenging at this stage compared with that at 710–760 °C.

Next, a scheme for decreasing reactivity by increasing the *x* in NMC622 is proposed. On increasing *x*, the proportion of Ni^2+^ in the bulk and on the surface reduces along with the Li deficiency and the number of oxygen vacancies. This contributes toward a reduction in cation mixing in the initial state. In addition, the Li that does not dissolve in NMC622 forms a Li_2_CO_3_‐like surface‐coating layer on the particle surface, which decomposes into an Li_2_O surface layer at ≈ 680 °C. These effects suppress the movement of Ni^2+^ between NMC622 and LLZ‐CaBi and prevent the formation of impurity phases. With increasing *x*, cation mixing decreases, and the formation of the Li_2_CO_3_‐like layer increases. An excessive increase in *x* causes the NMC622 and LLZ‐CaBi to react. When the Li_2_CO_3_‐like phase melts, an increase in the amount of liquid phase likely activates the movement of atoms through the liquid phase; details of this phenomenon require further investigation.

### 2.4 Characteristics of a Co‐Sintered Bulk‐Type ASSB Comprising NMC622 and LLZ‐CaBi

**Figure 12:**
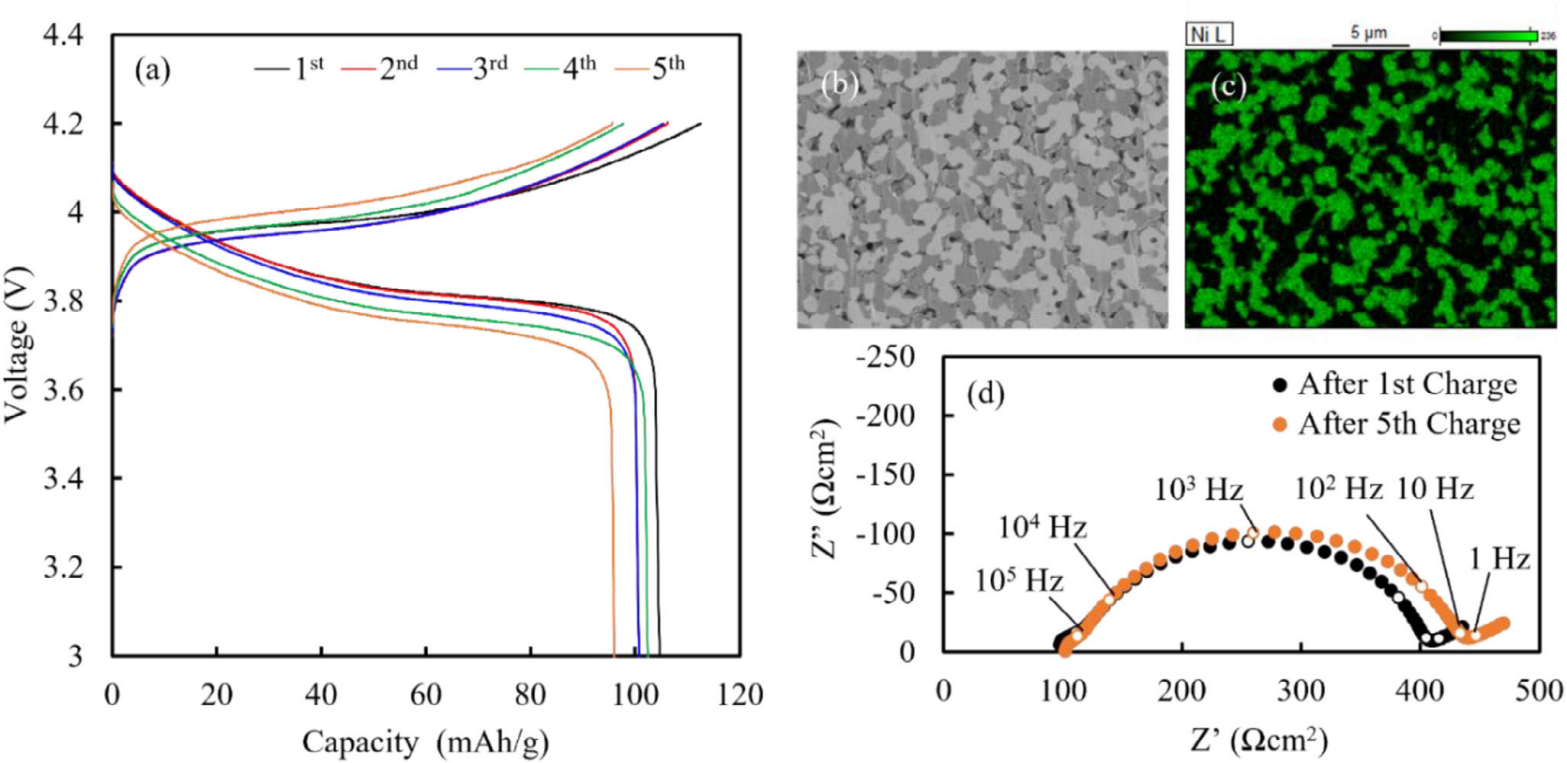
a) Charge–discharge curves, b) cross‐sectional scanning electron microscopy image, c) Ni mapping, and(d) Nyquist plots of the Li|PEO|LLZ‐CaBi|Li_10.75_Ni_0.6_Mn_0.2_Co_0.2_O_2_+LLZ‐CaBi all‐solid‐state battery at 0.1C and 60 °C.

Li_1+_ *_x_* Ni_0.6_Mn_0.2_Co_0.2_O_2_ and LLZ‐CaBi do not form impurity phases when sintered at 800 °C; therefore, they can be co‐sintered. The density and ionic conductivity of LLZ‐CaBi sintered at 750 and 800 °C are shown in Figure S11 (Supporting Information). Increasing the sintering temperature to 800 °C improves the relative density but reduces the ionic conductivity of the system. Moreover, an increase in the grain boundary resistance causes a reduction in conductivity, likely due to the effect of active dissolution and precipitation at the LLZ‐CaBi/Li–Ca–Bi–O interface on the abundant formation of Li_2_ZrO_3_ at the grain boundaries. This phenomenon can be controlled by tuning the initial composition; however, the modulation of this phenomenon is beyond the scope of this study and will be reported in the future. LLZ‐CaBi sintered at 800 °C exhibits high density and ionic conductivity, making it a useful electrolyte for bulk‐type ASSBs. Cross‐sectional SEM–EDX images of the fabricated ASSB are shown in **Figure** **12**b,c. In Figure 12b, the grey, white, and black areas represent NMC622, LLZ‐CaBi, and pores, respectively. Compared with a previously reported cathode layer comprising LiCoO_2_, the cathode layer analyzed here contains multiple pores and a low density, likely owing to the particle size of the active material. According to the literature, LLZ‐CaBi shows low stability against Li metal; therefore, this material is only suitable for cathodes. Therefore, polyethylene oxide (PEO) was placed on the separator and the system was evaluated at 60 °C. The charge–discharge test results of the fabricated ASSB are shown in Figure 12a. The fabricated ASSB can be repeatedly charged and discharged. This is the first study to report a repetitively operable ASSB comprising NMC622 and LLZ manufactured by co‐sintering through conventional ceramics processing. The 1st charge capacity of the system (113 mAh g^−1^ (0.29 mAh cm^−2^)) is 68% the theorical value, and the initial discharge capacity decreases by 8.3% after five cycles. Notably, the capacity of a previously reported ASSB manufactured by co‐sintering LiCoO_2_ and LLZ‐CaBi decreases rapidly after ≈10 cycles, consistent with the results of this study.^[^ ^30^ ^]^ Figure 12d shows the Nyquist plot of the manufactured system after 1st charging and 5th charging; two distinct semicircles are observed in the frequency range above 10 Hz. The resistance increases by ≈ 10–100 Hz after cycling. According to Akimoto et al., the interface resistance between NMC111 and LLZ is <80 Hz.^[^ ^26^ ^]^ Therefore, the capacity fading observed in this study can be attributed to the increase in interface resistance between NMC622 and LLZ‐CaBi during cycling. However, the detailed mechanism underlying this increment requires further analysis. **Table** **1** summarizes the characteristics and degradation factors of certain all‐solid‐state batteries using sulfide‐, halide‐, and oxide‐based electrolytes. Although sulfide‐based electrolytes exhibit high ionic conductivity, they are prone to decomposition due to weak against oxidation potential, which accelerates battery degradation.^[^ ^64^, ^65^, ^66^ ^]^ To prevent this, it is being considered to coat the surface of the cathode active material with an oxide such as Li‐Zr‐O.^[^ ^64^ ^]^ However, the coating is not sufficient to avoid the decomposition of the electrolyte material. Additionally, the loss of contact between materials due to the expansion and contraction of the active material promotes deterioration. In contrast, halide‐ and oxide‐based electrolytes are stable, even at oxidation potentials; in such systems, cracks (likely attributable to active‐material expansion) at various locations are a cause of deterioration.^[^ ^67^, ^68^, ^69^, ^70^, ^71^ ^]^ Notably, NMC622 undergoes a volume change of ≈1.2% during charging and discharging,^[^ ^72^ ^]^ likely resulting in the disruption of Li‐ion pathways at the NMC622/LLZ interface. In future research, we plan to evaluate the long‐term cycle performance and rate characteristics of this battery and investigate the factors affecting its degradation.

**Table 1:** A comparison of the degradation factors for ASSBs using sulfide‐, halide‐, and oxide‐based electrolytes.

Electrolyte type	Reference	Cathode composition	Voltage range [V]	Number of cycles	Capacity retention rate	Degradation factor
Sulfide	Shi et al. (2020)^[^ ^64^ ^]^	LiNi_0.5_Mn_0.3_Co_0.2_O_2_ +75Li_2_S‐25P_2_S_5_		50	15.5%	Oxidative decomposition of the electrolyte. Loss of contact at the active material/electrolyte interface
	Koerver et al. (2017)^[^ ^65^ ^]^	LiNi_0.8_Mn_0.1_Co_0.1_O_2_ +β‐Li_3_PS_4_	2.7–4.3	50	65%	
	Yoshinari et al. (2019)^[^ ^66^ ^]^	LiNi_0.8_Mn_0.1_Co_0.1_O_2_ +β‐Li_3_PS_4_	2.65–4.35	50	51％	
	Zhang et al. (2018) ^[^ ^67^ ^]^	LiCoO_2_+Li_10_GeP_2_S_12_	2.6–4.2	300	62.5%	
Halide	Yun et al. (2023)^[^ ^68^ ^]^	LiNi_0.8_Mn_0.1_Co_0.1_O_2_ +Li_3_InCl_6_	2.5–4.3	100	75.7%	Cracks in the electrolyte material
	Han et al. (2021)^[^ ^69^ ^]^	LiNi_0.88_Co_0.11_Al_0.01_O_2_+Li_3_YCl_6_	3–4.3	200	74%	Cracks in the active material. Loss of contact at the active material/electrolyte interface
Oxide	Guo et al. (2021)^[^ ^70^ ^]^	LiCoO_2_+ Li_6.75_La_3_Zr_1.75_Nb_0.25_O_12_	2.8–4.35	30	65.8%	Cracks between the positive electrode layer and the separator layer
	Tsai et al. (2018) ^[^ ^71^ ^]^	LiCoO_2_+ Li_6.6_La_3_Zr_1.6_Ta_0.4_O_12_	2.65–4.35	100	30%	Cracks in the active and electrolyte materials. Loss of contact at the active material/electrolyte interface

## 3 Conclusion

In summary, the results of this study confirm that Li_1+_ *_x_* Ni_0.6_Mn_0.2_Co_0.2_O_2_, synthesized with a Li‐rich composition rather than a stoichiometric composition, is suitable for co‐sintering with LLZ. At *x* = 0.075 and sintered at 800 °C, no impurity phase is formed when Li_1+_ *_x_* Ni_0.6_Mn_0.2_Co_0.2_O_2_ is co‐sintered with LLZ‐CaBi. Typically, the formation of an impurity phase occurs between NMC622 and LLZ‐CaBi due to the migration of Ni^2+^ ions. In the high‐temperature sintering environment, the transition metals (Ni, Mn, and Co) in the synthesized system exist in a low‐valence state, thereby undergoing facile cation mixing. Increasing the *x* reduces cation mixing because the Ni in the synthesized NMC remains in a high‐valence state. In addition, a Li_2_CO_3_‐like layer is formed on the surface of the NMC622 particles, preventing the diffusion of Ni into LLZ‐CaBi. Interestingly, the developed NMC622 can be used to fabricate a bulk‐type ASSB with excellent charge–discharge performance without interfacial‐layer addition using conventional co‐sputtering. The method proposed in this study is expected to guide future research on the design and development of high‐capacity systems through co‐sintering with NMC materials (including NMC811 and NMC955).

## 4 Experimental Section

### Synthesis of NMC622

Li_1+_ *_x_* Ni_0.6_Mn_0.2_Co_0.2_O_2_ (*x* = 0.01, 0.025, 0.05, 0.075, 0.1, 0.15, or 0.2) powders were synthesized using the amorphous malic‐acid precursor method, which is similar to a previously reported Li_1+_ *_x_* CoO_2_ synthesis method.^[^ ^46^ ^]^ First, C_4_H_6_O_5_ (99%; Fujifilm Wako Pure Chemical Corp., Japan), LiNO_3_ (99%; Fujifilm Wako Pure Chemical Corp., Japan), Ni(NO_3_)_2_·6H_2_O (98%; Fujifilm Wako Pure Chemical Corp., Japan), Mn(NO_3_)_2_·6H_2_O (98%; Fujifilm Wako Pure Chemical Corp., Japan), and Co(NO_3_)_2_·6H_2_O (99.5%; Fujifilm Wako Pure Chemical Corp., Japan) were dissolved in distilled water. After adjusting the pH of the mixed solution to 3.0 using aqueous ammonia (28%), the solution was evaporated to dryness and heated to 400 °C until reactive ignition was unobservable. The resultant powder was calcined at 850 °C for 10 h and ground in 2‐propanol in a uniaxial ball mill with zirconia balls.

### Synthesis of LLZ‐CaBi

Li_6.5_(La_2.79_Ca_0.08_)(Zr_1.42_Bi_0.58_)O_12_ samples were synthesized by a solid‐state reaction. LiOH(H_2_O) (>99.95%; Sigma–Aldrich, Germany), La(OH)_3_ (>99.99%; Kojundo Chemical Laboratory Co., Ltd., Japan), ZrO_2_ (>98%; Kojundo Chemical Laboratory Co., Ltd., Japan), Ca(OH)_2_ (>99.9%; Kojundo Chemical Laboratory Co., Ltd., Japan), and Bi_2_O_3_ (>99.999%; Kojundo Chemical Laboratory Co., Ltd., Japan) were used as starting materials. These materials were weighted and ball‐milled in 2‐propanol in a uniaxial ball mill using zirconia balls of 2‐mm diameter. After solvent evaporation, the powders were calcined in an alumina crucible at 800 °C for 12 h in dry air. The resulting powders were ground in 2‐propanol in a uniaxial ball mill using zirconia balls and pressed at 98 MPa to form disks, which were sintered at 750 and 800 °C for 24 h in dry air. To avoid Al contamination from the alumina setter, the pellets were placed on Au sheets.

### NMC622/LLZ‐CaBi Reactivity Analysis

The NMC622 and LLZ‐CaBi powders were mixed in a ZrO_2_ mortar at a volume ratio of 50:50. The synthesized powders were pressed at 98 MPa to form disks, which were sintered at 750 and 800 °C for 24 h on Au sheets. After sintering, the pellets were crushed in a ZrO_2_ mortar and analyzed by XRD. The XRD measurement conditions are detailed in the relevant section.

### Characterization of Material

XRD measurements acquired on a SmartLab (Rigaku, Japan) with Cu *K*α radiation were used for phase identification in the synthesized powders. PDXL (Rigaku, Japan) was used for analysis.

Field‐emission scanning electron microscopy with a JEM‐6510 microscope (JEOL, Japan) was used to assess the sample microstructure. The elemental distribution was probed using EDX (QUANTAX Flat QUAD System Xflash 5060FQ, Bruker, Germany). The samples were polished via ion milling using an IB‐19520CCP instrument (JEOL, Japan). STEM with a JEM‐ARM200F (JEOL, Japan) was used to assess their ultrafine microstructure. Thin samples were fabricated using a focused ion beam with a NanoDUET NB5000 instrument (Hitachi High‐Tech Ltd., Japan). Elements were detected with EDX and EELS (GIF Quantum‐ER, Gatan Inc., Japan).

Quantitative elemental analysis was conducted using ICP‐AES on an Agilent 5110 VDV instrument (Agilent Technologies, USA).

The thermal decomposition of the powders was evaluated by GC–MS using a JMS‐Q1600GC instrument (JEOL, Japan). The samples were heated to 900 °C at a constant rate of 10 °C min^−1^ in a He atmosphere.

Relative densities were calculated from the pellet size, weight, and theoretical densities of the powders. Here, theoretical densities were evaluated by a gas‐displacement pycnometer system using an AccuPycII 1340 (SHIMADZU Corp., Japan).

XAFS measurements were conducted on beamline BL‐9C at KEK (Tsukuba, Japan). A Cu foil was used for the energy calibration of the monochromator. The temperature was changed from 25 to 900 °C under nitrogen and oxygen flow (at 40 and 10 cc min^−1^, respectively). After setting a heating rate of 5 °C min^−1^, measurements were recorded in the transmission mode. EXAFS spectra were analyzed using Artemis (Open software). The fitting range was *k* = 3–15 Å^−1^ and *R* = 1.15–1.9 Å at fixed values of $S_{0}^{2}$ (= 0.82) and *E*_0_ (= 2.79 eV).

### Electrical Characterization

The Li‐ion conductivity of the manufactured pellets was determined using Li‐blocking Au‐sputtered electrodes with a two‐probe AC impedance method using a 4990EDMS (Keysight Technologies, USA) in the frequency range of 20 Hz–100 MHz at 298K. Before Au sputtering, the surface of each sample was polished well with emery paper to remove the impurity phase, and the sample thickness was set to 0.8–1.0 mm.

### Bulk‐Type ASSB Performance

Powders of NMC622 (*x* = 0.075) and LLZ‐CaBi were mixed in a volume ratio of 50:50 for the composite cathode. The mixed powder (2.5 mg; final loading amount after sintering is 6.5 mg cm^−2^) was laminated onto a separator layer comprising LLZ‐CaBi (0.18 g) in a Φ8 mold and compacted at 98 MPa. The green compact was sintered at 800 °C for 24 h to form a half cell. Subsequently, the cathode and separator layers were sintered together. After sintering, Au was sputtered onto the cathode surface as the current collector. Li foil was used as the negative electrode, and a PEO‐based solid polymer electrolyte was inserted as a buffer layer. PEO was fabricated according to a previously reported method.^[^ ^28^ ^]^ Cycle testing was carried out at 0.1C (21 µA: 54 µA cm^−2^) for 5 cycles at 60 °C using a potentio‐galvanostat 1287A (Solartron Analytical, U.K.). The high and low cutoff voltages were set to 4.2 and 3.0 V, respectively. AC electrochemical impedance spectroscopy was carried out within 10^6^–0.1 Hz using a frequency response analyzer (1260A; Solartron Analytical, U.K.).

## Conflict of Interest

The authors declare no conflict of interest.

## Supporting information



Supporting Information

## Data Availability

The data that support the findings of this study are available from the corresponding author upon reasonable request.
