# Supplementary material for: Reaction Suppression Between a High‐Ni Cathode Material (NMC622) and Li7La3Zr2O12 on Co‐Sintering for Manufacturing Bulk‐Type All‐Solid‐State Batteries: A New Method and Its Mechanism
Source: Adv Sci (Weinh). 2025 Aug 29;12(43):e12219. doi: 10.1002/advs.202512219 (PMC12631915; doi:10.1002/advs.202512219)
Supplement: Supplementary file 1 — Supporting Information [file ADVS-12-e12219-s001.docx]

Supporting Information

Reaction Suppression Between a High-Ni Cathode Material (NMC622) and Li_7_La_3_Zr_2_O_12_ on Co-Sintering for Manufacturing Bulk-type All-Solid-State Batteries: A New Method and its Mechanism

Naohiro Hayashi*, and Ken Watanabe*

**Table S1.** ICP results for the Li_1+_*_x_*Ni_0.6_Mn_0.2_Co_0.2_O_2_ particles. The molar composition ratio is calculated based on Co.

|  | Weight composition ratio (wt%) | | | | Molar composition ratio (mol%) | | | |
| --- | --- | --- | --- | --- | --- | --- | --- | --- |
| *x* in Li_1+x_Ni_0.6_Mn_0.2_CoO_2_ | Li | Ni | Mn | Co | Li | Ni | Mn | Co |
| 0.01 | 6.72 | 34.3 | 11.1 | 11.6 | 0.98 | 0.59 | 0.21 | 0.2 |
| 0.025 | 6.86 | 35.4 | 11.4 | 11.9 | 1.00 | 0.60 | 0.21 | 0.2 |
| 0.050 | 6.87 | 34.5 | 11.2 | 11.5 | 1.02 | 0.60 | 0.21 | 0.2 |
| 0.075 | 6.96 | 34.1 | 11.0 | 11.5 | 1.02 | 0.60 | 0.21 | 0.2 |
| 0.10 | 7.00 | 34 | 11.0 | 11.4 | 1.02 | 0.60 | 0.21 | 0.2 |
| 0.15 | 7.05 | 33.7 | 10.8 | 11.3 | 1.03 | 0.60 | 0.21 | 0.2 |
| 0.20 | 7.22 | 33.7 | 10.8 | 11.3 | 1.06 | 0.60 | 0.21 | 0.2 |

**
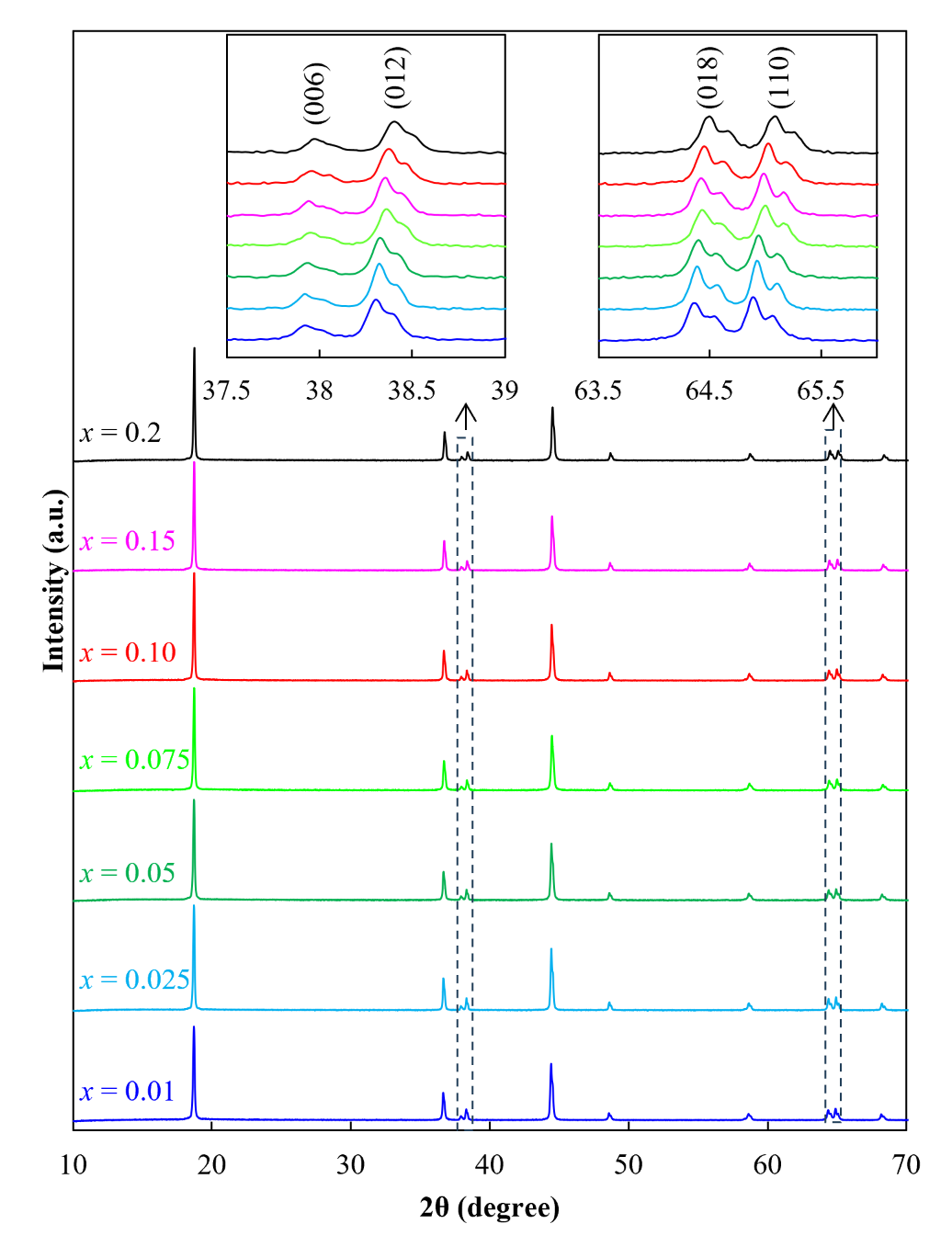
**

**Figure S1.** XRD patterns of the Li_1+_*_x_*Ni_0.6_Mn_0.2_Co_0.2_O_2_ powder.

**Table S2.** Rietveld refinement results of the the Li_1+_*_x_*Ni_0.6_Mn_0.2_Co_0.2_O_2_ powder.

|  | Lattice parameter | | | R factor | | |
| --- | --- | --- | --- | --- | --- | --- |
| *x* in Li_1+x_Ni_0.6_Mn_0.2_CoO_2_ | a (Å) | b (Å) | ｃ(Å) | R_wp_ (%) | R_p_ (%) | R_e_ (%) |
| 0.01 | 2.8722 | 2.8722 | 14.2269 | 2.26 | 1.62 | 1.58 |
| 0.025 | 2.8704 | 2.8704 | 14.2212 | 2.13 | 1.54 | 1.58 |
| 0.050 | 2.8670 | 2.8670 | 14.2202 | 2.21 | 1.59 | 1.58 |
| 0.075 | 2.8679 | 2.8679 | 14.2129 | 2.41 | 1.72 | 1.60 |
| 0.10 | 2.8672 | 2.8672 | 14.2119 | 2.33 | 1.56 | 1.58 |
| 0.15 | 2.8666 | 2.8666 | 14.2107 | 2.30 | 1.70 | 1.59 |
| 0.20 | 2.8643 | 2.8643 | 14.2056 | 2.55 | 1.79 | 1.59 |

**Table S3.** Molar amount of Li_2_CO_3_ contained in Li_1+_*_x_*Ni_0.6_Mn_0.2_Co_0.2_O_2_.

| *x* in Li_1+x_Ni_0.6_Mn_0.2_CoO_2_ | Molar amount of Li_2_CO_3_ (mol%) |
| --- | --- |
| 0.025 | 1.36 |
| 0.050 | 1.44 |
| 0.075 | 1.55 |
| 0.10 | 1.57 |
| 0.15 | 1.97 |
| 0.20 | 2.59 |


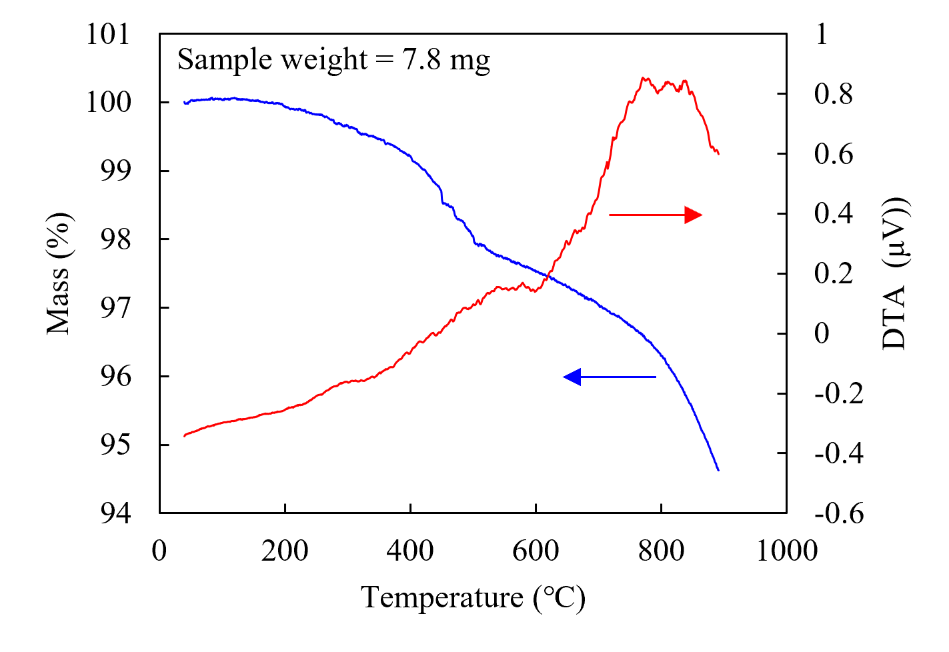


**Figure S2.** TG–DTA results for the Li_1+_*_x_*Ni_0.6_Mn_0.2_Co_0.2_O_2_ powder.

**
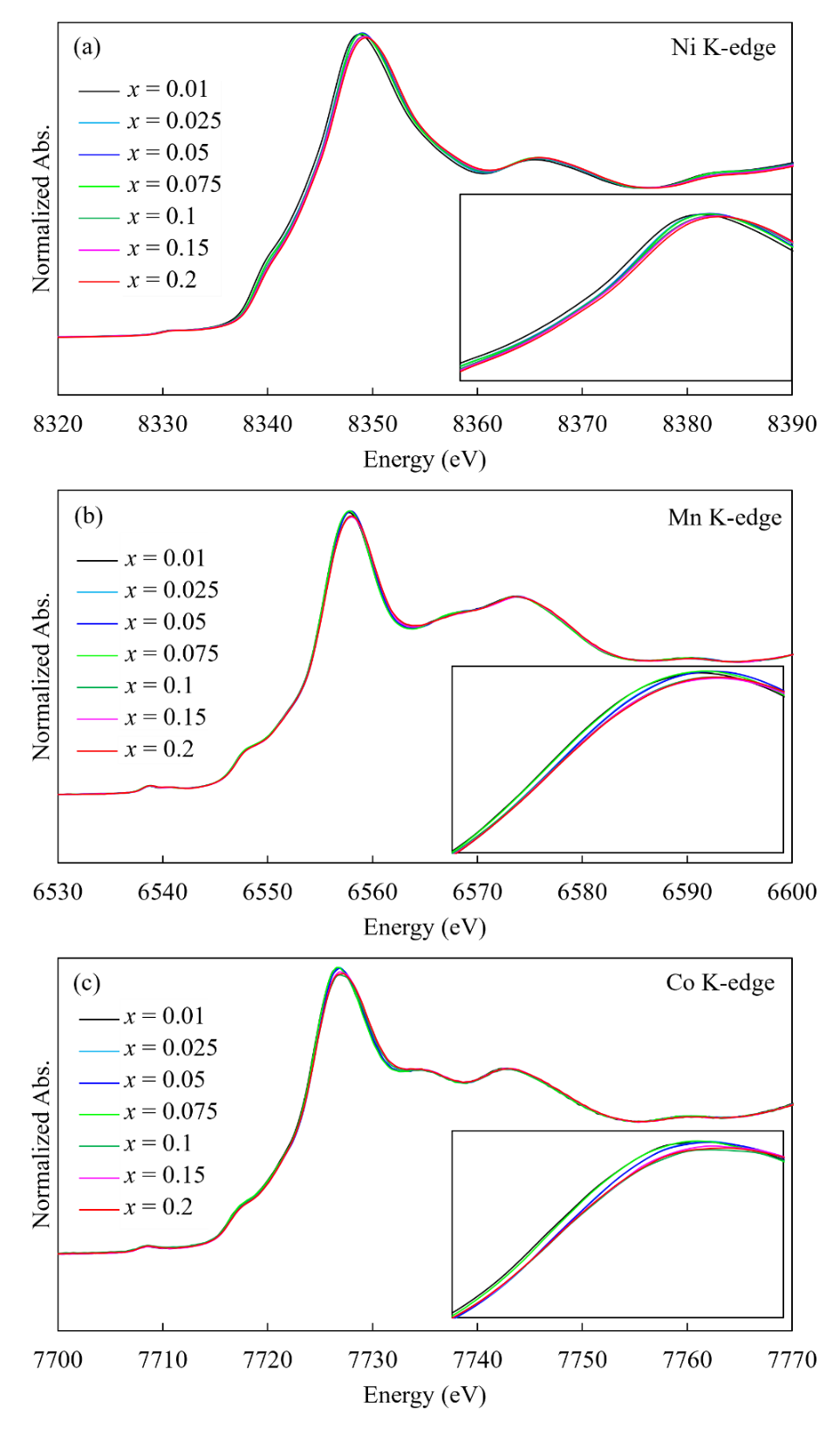
**

**Figure S3.** Normalized XANES spectra of Li_1+_*_x_*Ni_0.6_Mn_0.2_Co_0.2_O_2_ at the (a) Ni, (b) Mn, and and (c) Co K-edges.

**
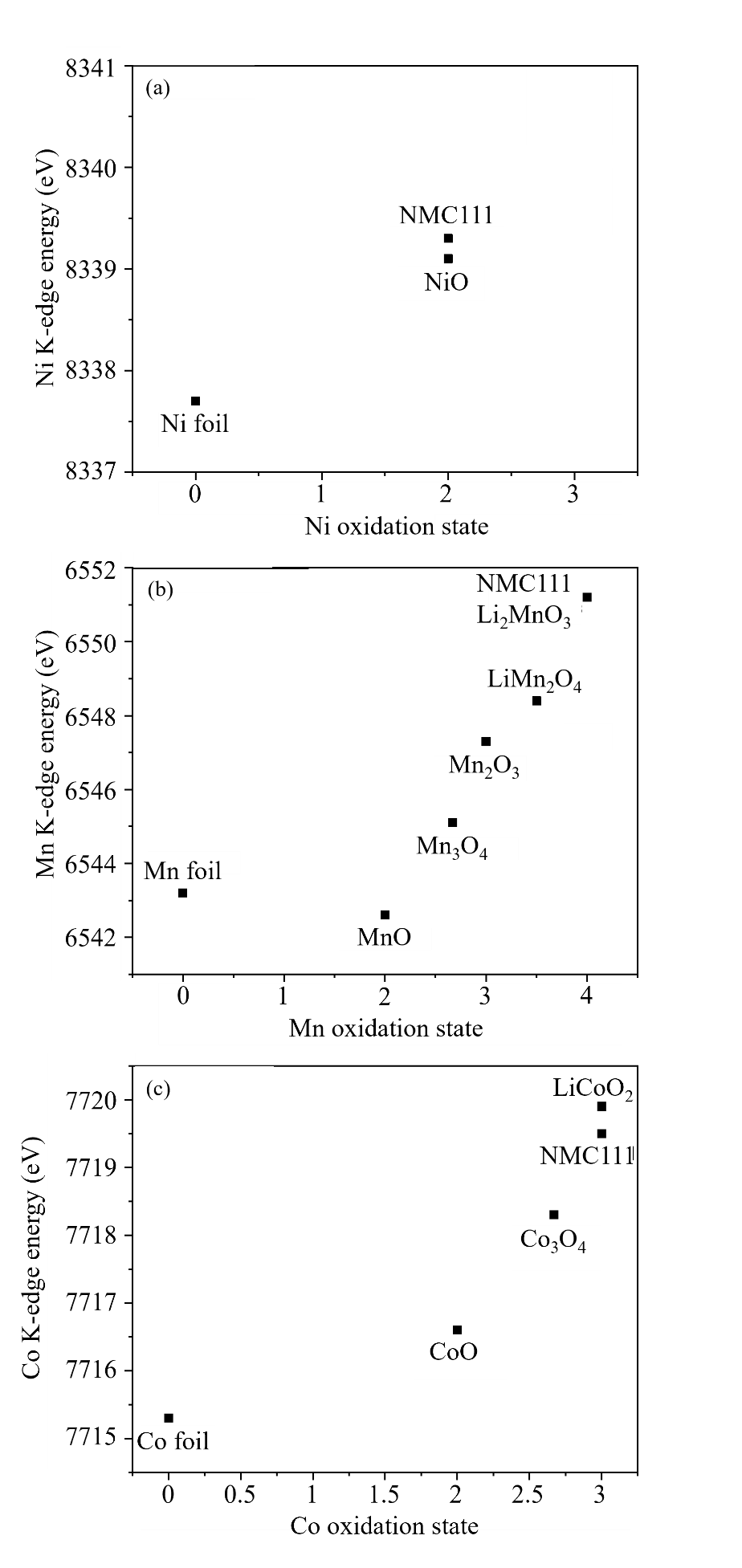
**

**Figure S4.** (a) Ni, (b) Mn, and (c) Co K-edge energies of reference samples.


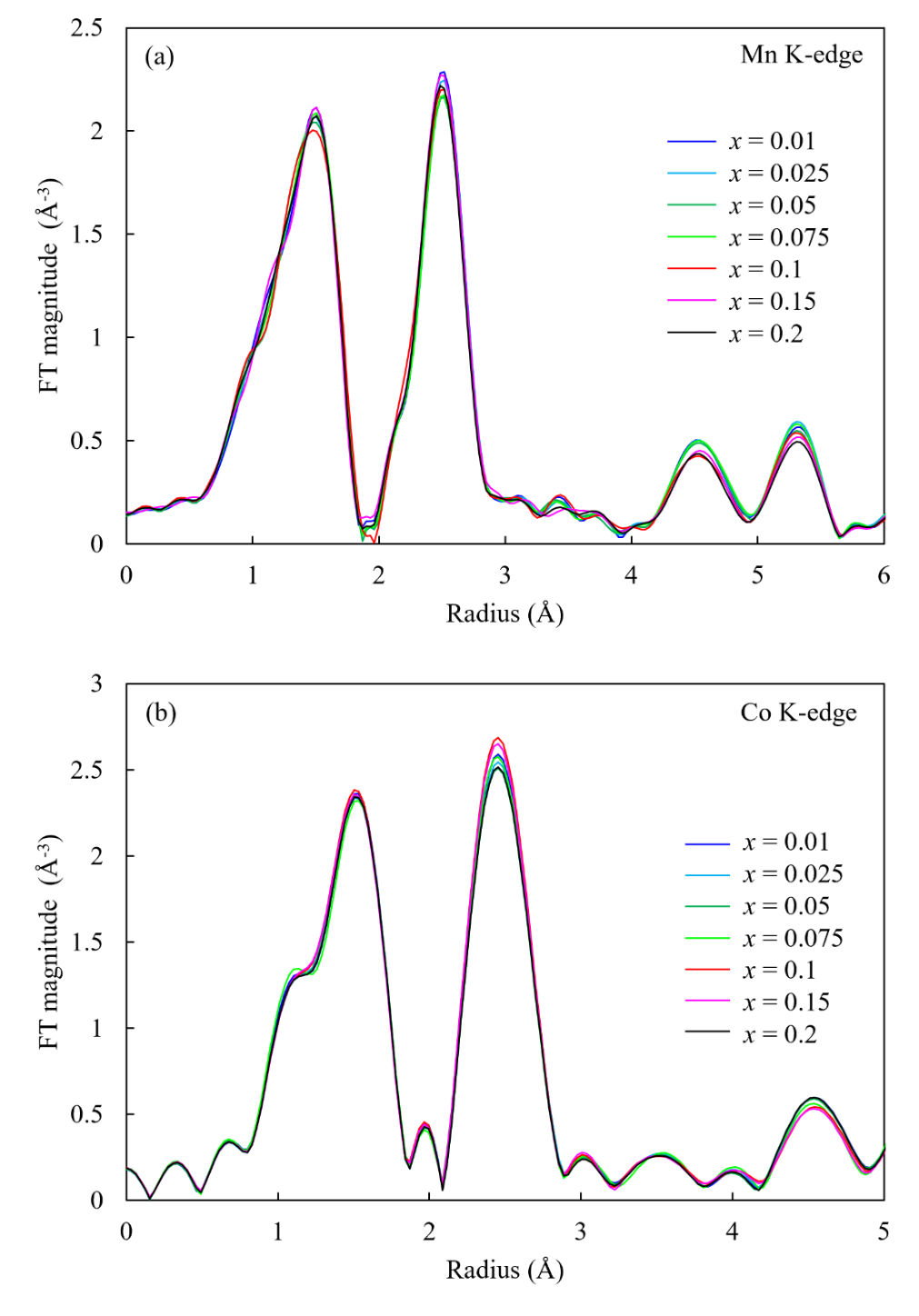


**Figure S5.** EXAFS (a) Mn, and (b) Co K-edge spectra for Li_1+_*_x_*Ni_0.6_Mn_0.2_Co_0.2_O_2_.


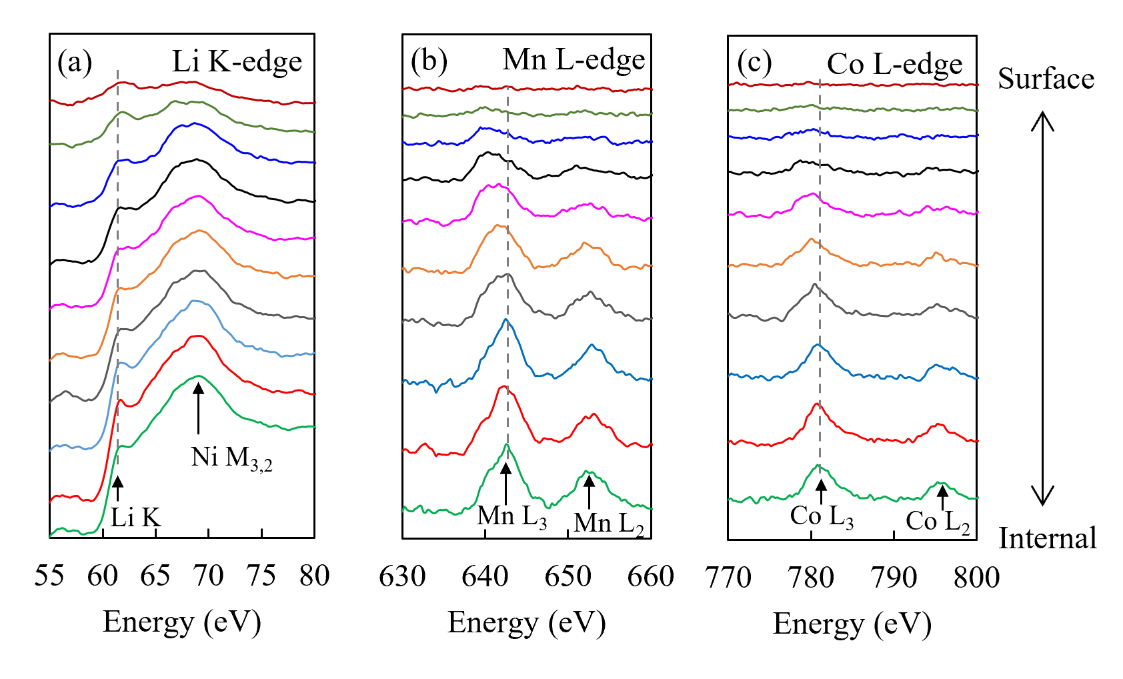


**Figure S6.** (a) Li K-edge, (b) Mn L-edge, and (c) Co L-edge EELS spectra of Li_1.01_Ni_0.6_Mn_0.2_Co_0.2_O_2_ particles (from the surface to the interior).


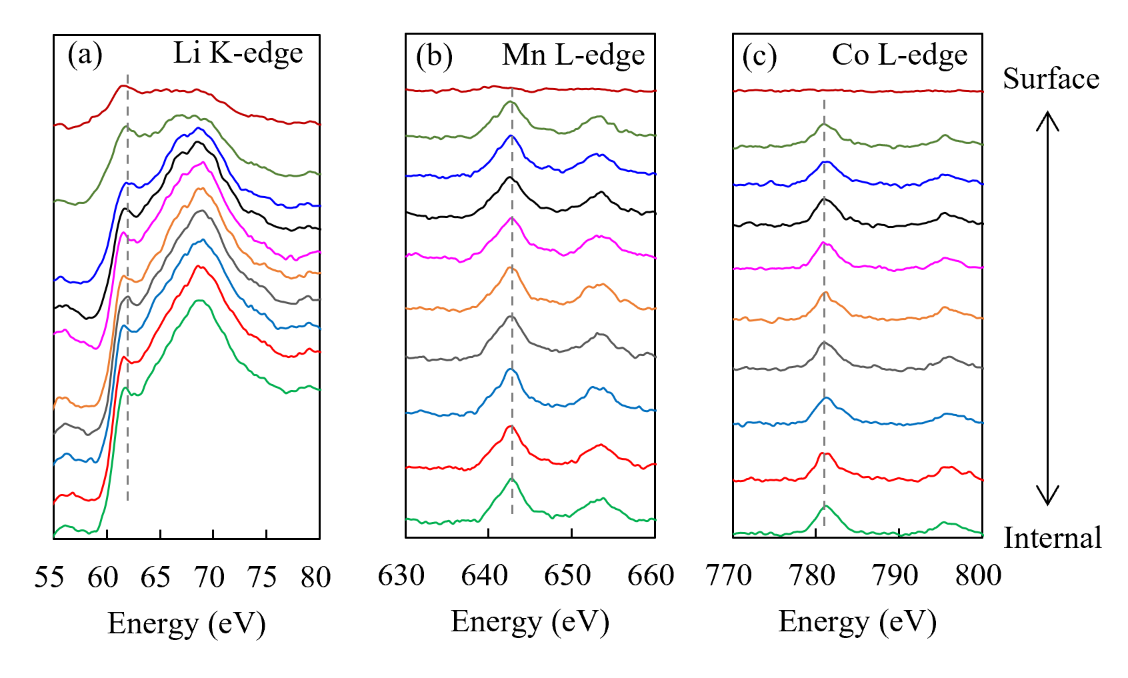


**Figure S7.** (a) Li K-edge, (b) Mn L-edge, and (c) Co L-edge EELS spectra of Li_1.075_Ni_0.6_Mn_0.2_Co_0.2_O_2_ particles (from the surface to interior).

**Table S4.** The I(003)/I(004) ratio calculated from Figure 1(a) and the amount of impurities calculated from Figure 3.

| *x* in NMC622 | I(003)/I(004) | Impurity ratio at 750 ℃ sintering | | Impurity ratio at 800 ℃ sintering | |
| --- | --- | --- | --- | --- | --- |
|  |  | Li_2_ZrO_3_ | La_2_(Li_0.5_Ni_0.5_)O_4_ | Li_2_ZrO_3_ | La_2_(Li_0.5_Ni_0.5_)O_4_ |
| 0.01 | 1.557 | 6.2 | 22.0 | 2.1 | 12.6 |
| 0.025 | 1.604 | 1.6 | 4.4 | 0.1 | 0.3 |
| 0.050 | 1.655 | 0.0 | 5.9 | 0.0 | 0.2 |
| 0.075 | 1.737 | 0.0 | 4.8 | 0.0 | 0.0 |
| 0.10 | 1.781 | 0.1 | 6.0 | 0.0 | 0.0 |
| 0.15 | 1.841 | 0.2 | 7.3 | 0.0 | 7.0 |
| 0.20 | 1.935 | 0.0 | 13.9 | 0.0 | 5.9 |

**
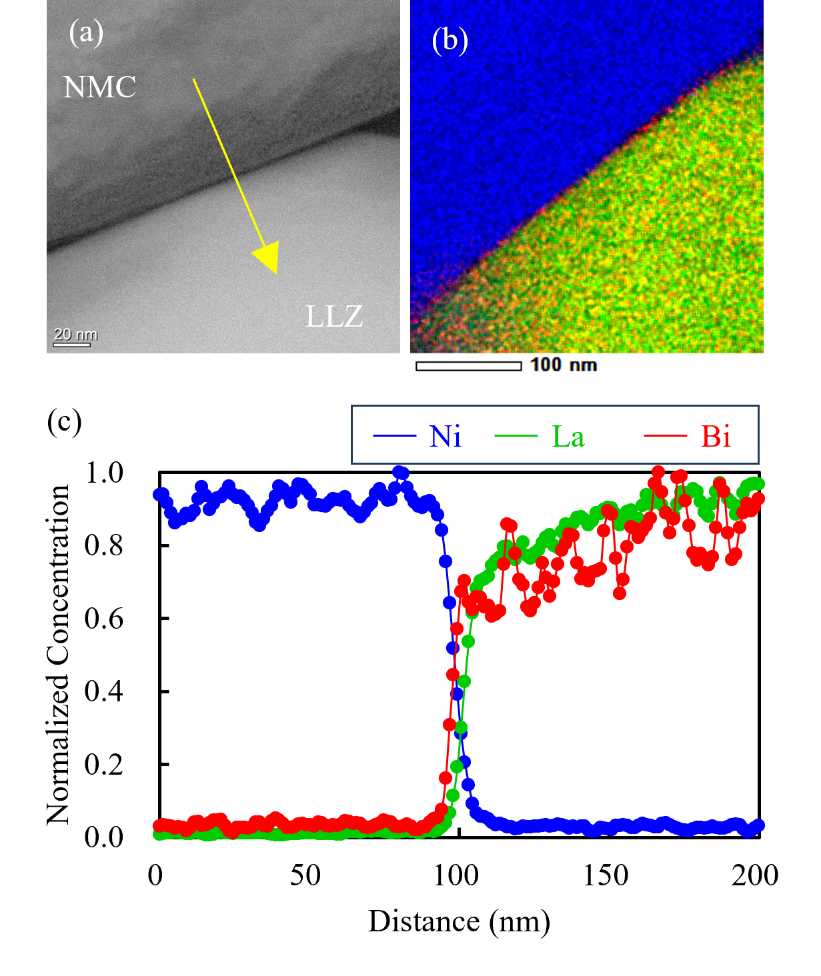
**

**Figure S8.** (a) STEM-HAADF image, (b) EDX superposition mapping (blue: Ni, green: La, red: Bi), and (c) element concentration profiles for the NMC622 (*x* = 0.01)/LLZ-CaBi interface produced by sintering at 800 ℃.


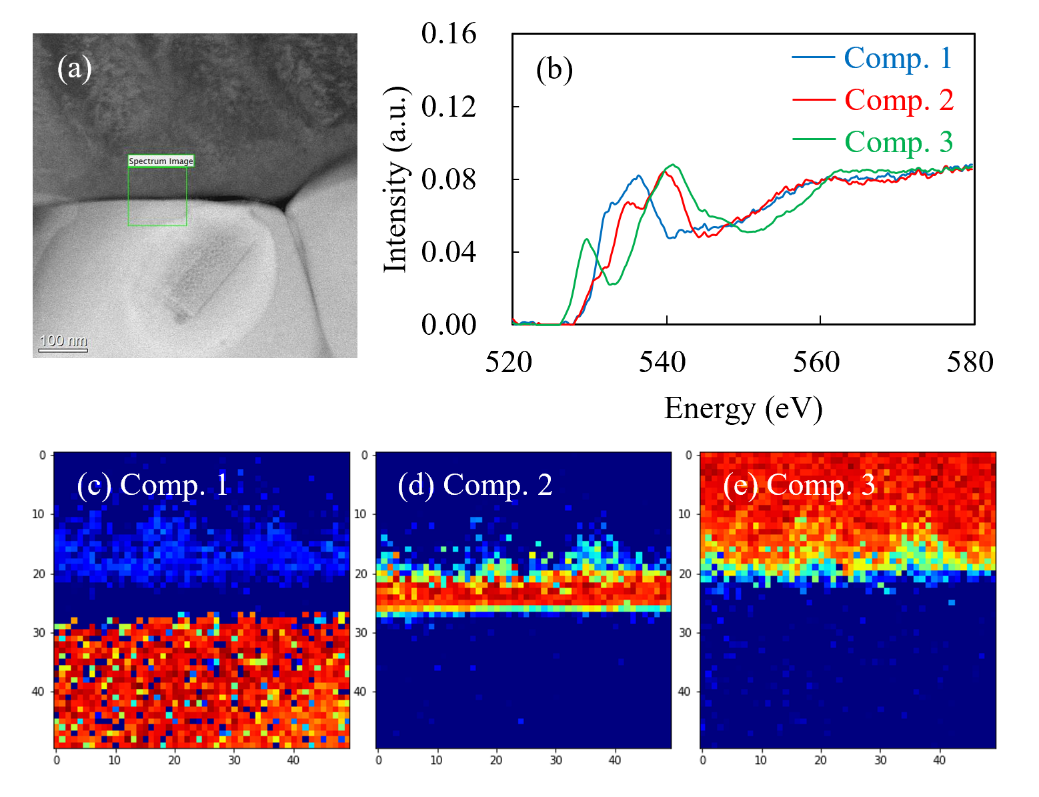


**Figure S9.** (a) STEM-HAADF image, (b) spectrum after decomposition by multivariate analysis, and (c)–(e) imaging results for each component of the NMC622 (*x* = 0.01)/LLZ-CaBi interface produced by sintering at 800 ℃.


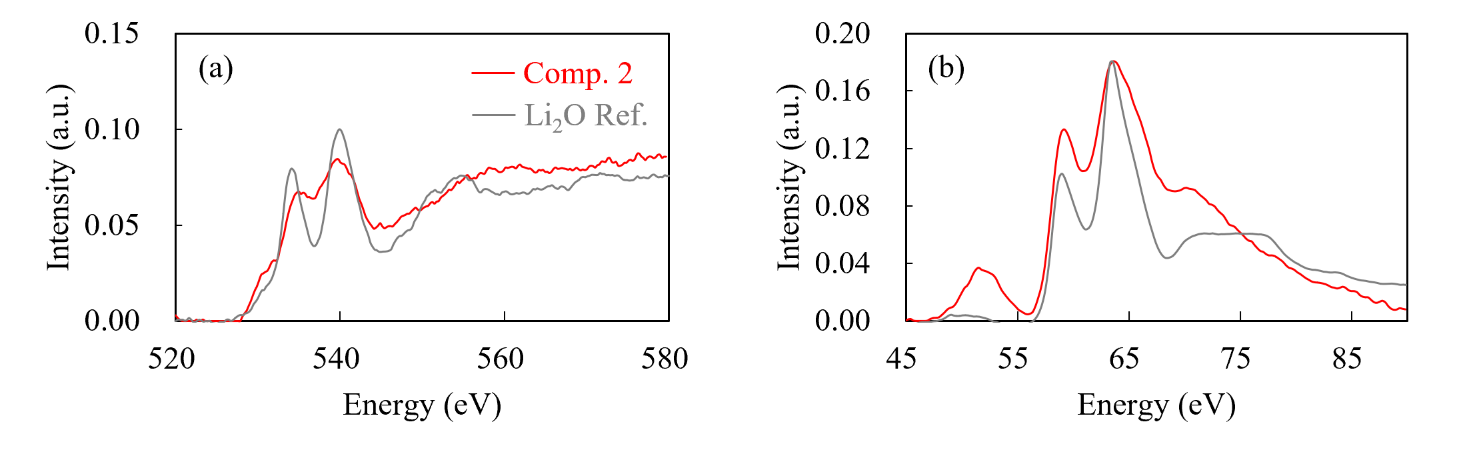


**Figure S10.** Comparison of the (a) O and (b) Li K-edge data for component 2 with Li_2_O reference data.


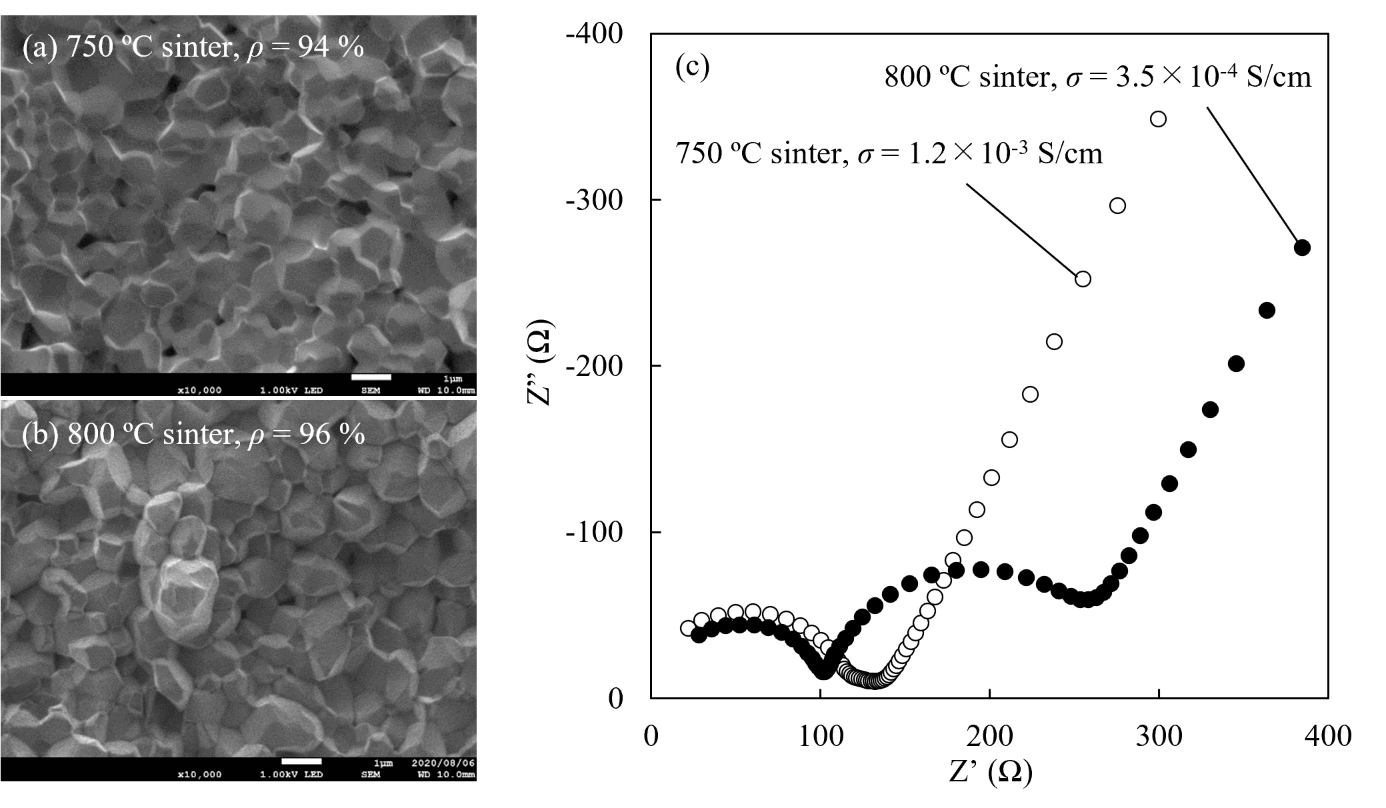


**Figure S11.** (a), (b) Fracture surface images, relative densities, (c) Nyquist plots and Li-ion conductivity of Li_6.5_(La_2.79_Ca_0.08_)(Zr_1.42_Bi_0.58_)O_12_ pellets sintered at (a) 750 and (b) 800 ℃.
